# A Facile Strategy to Restore the Optic Nerve Functionality Using an Injectable Conducting Hydrogel

**DOI:** 10.1002/advs.202415601

**Published:** 2025-04-17

**Authors:** Changchun Yu, Yandi Zhou, Shuang Yao, Ziyi Wang, Sihao Ye, Rubing Qi, Hai Hu, Keke Liu, Yabo Wu, Tom Lawson, Lu Yan, Yong Liu, Wencan Wu

**Affiliations:** ^1^ School of Ophthalmology and Optometry School of Biomedical Engineering Wenzhou Medical University Wenzhou Zhejiang 325027 China; ^2^ National Engineering Research Center of Ophthalmology and Optometry Eye Hospital Wenzhou Medical University Wenzhou Zhejiang 325027 China; ^3^ Zhejiang Key Laboratory of Key Technologies for Visual Pathway Reconstruction Wenzhou Medical University Wenzhou Zhejiang 325000 China; ^4^ Oujiang Laboratory (Zhejiang Laboratory for Regenerative Medicine Vision and Brain Health) Wenzhou Zhejiang 325000 China; ^5^ School of Biomedical Engineering and Imaging Sciences King's College London London WC2R2LS UK; ^6^ School of Chemical Engineering University of New South Wales Sydney NSW 2052 Australia

**Keywords:** nerve regeneration, optic nerve injuries, poly(3,4‐ethylenedioxythiophene) (PEDOT), retinal ganglion cells, vision functionality repair

## Abstract

This study introduces a novel injectable conductive polymer hydrogel from poly(3,4‐ethylenedioxythiophene) (PEDOT). This hydrogel is designed to facilitate the recovery of electrophysiological function in injured optic nerves. The hydrogel can be injected directly at the injury site and spontaneously gel in place. These findings indicate a remarkable restoration of electrophysiological function, with a two to fourfold increase in amplitude (N1‐P1 wavelet) of flash visual evoked potentials. Electroretinography results further support that both a‐wave and b‐wave amplitudes obtained under various adaptation conditions in the PEDOT hydrogel‐treated animal models with optic nerve crush are comparable to those in the control group. The visual cliff testing results further supports the enhanced visual functionality after the hydrogel treatment. This improvement signifies enhanced responsiveness to light stimulation and a substantial boost in axonal transmission of visual information. Additionally, the injection of PEDOT hydrogel significantly mitigates the apoptosis of retinal ganglion cells, resulting in a fourfold increase in their survival rate. The ease of preparation and outstanding performance in vivo make it a promising bioengineered material for optic nerve research. This advancement offers new hope for patients with traumatic optic neuropathy, potentially leading to improved treatments and outcomes in optic nerve regeneration.

## Graphical abstract

An injectable conductive polymer hydrogel from poly (3,4‐ethylenedioxythiophene) (PEDOT) is developed to facilitate the recovery of electrophysiological function in injured optic nerve. The hydrogel can be injected directly at the injury site and spontaneously gel in place.

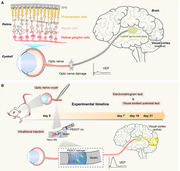

## 1 Introduction

The optic nerve, a key component of the central nervous system (CNS), serves as a conduit for transmitting information in the form of electrical impulse to support neuronal function, neurite growth, and nerve regeneration.^[^ ^1^ ^]^ However, when traumatic optic neuropathy results in axonotmesis (a structural injury to the axon with preservation of the surrounding connective tissue), it compromises the axonal compartment, leading to neurodegeneration.^[^ ^2^ ^]^ This disruption in the transmission of visual stimuli ultimately results in permanent vision loss, primarily due to the limited regenerative capacity.

One formidable challenge in the realm of optic nerve regeneration is the restoration of its function with connectivity to the brain. Injury‐induced calcium influx in the axoplasm can activate downstream effectors that, in turn, regulate the regeneration process.^[^ ^3^ ^]^ Unfortunately, this pathway is disrupted in injured nerves. To address this issue, nerve grafts (NGs) are employed as soft implants bridging the gap between two separated nerve stumps, thereby reconnecting the severed neural pathway.^[^ ^4^, ^5^ ^]^ These traditional NGs are typically composed of natural or synthetic materials, such as commercial collagen and chitosan products,^[^ ^6^ ^]^ polylactic acid,^[^ ^7^ ^]^ silk,^[^ ^8^, ^9^ ^]^ chitin,^[^ ^10^ ^]^ and others. These polymers have the capacity to deliver external neurotrophic factors and guide neurite growth at the site of nerve injury in therapeutic applications.^[^ ^11^ ^]^ However, they primarily serve as conduits to guide axonal regeneration and are unable to transmit electrical signals between the severed nerve ends.

Conductive NGs can replicate the high electrical transmission properties of native nerves, facilitating biosignal transduction.^[^ ^12^ ^]^ In contrast to conventional inorganic materials that mainly transport electrons and holes, organic conducting polymers, such as polypyrrole (PPy) and poly(3,4‐ethylenedioxythiophene) (PEDOT), employ a unique combination of ionic and electronic conduction mechanisms, enabling direct signal transduction.^[^ ^13^ ^]^ These materials have the potential to transmit endogenous electric signals and facilitate communication via electrical synapses, thereby enhancing tissue regeneration.^[^ ^14^ ^]^ For instance, a PPy‐coated nerve guidance conduit has demonstrated comparable performance to the autograft group, underscoring its promise for peripheral nerve regeneration.^[^ ^15^ ^]^ Similarly, PPy/silk fibroin conduits exhibit favorable properties for clinical applications, promoting nerve regeneration and functional recovery.^[^ ^16^ ^]^ These conjugated polymers share chemical characteristics with biological molecules and can be engineered into various forms, including hydrogels with Young's moduli similar to soft tissues,^[^ ^17^, ^18^ ^]^ potentially avoiding the triggering of injury‐response mechanisms that lead to scaffold encapsulation and nerve isolation.^[^ ^19^ ^]^

Conducting polymer hydrogels (CPHs) wherein electronic conductivity is introduced into the hydrogel structure with little compromise of the physical properties are of particular interest in tissue engineering.^[^ ^20^ ^]^ Hybrid PEDOT‐coated agarose hydrogels outperform plain hydrogel conduits in supporting neural regeneration.^[^ ^21^ ^]^ Collagen/PPy CPHs were found to enhance cell extension and neurogenesis.^[^ ^22^ ^]^ In the complex structure of the optic nerve, it is both risky and less effective to implant pre‐formed CPHs with specific cavity shapes as NGs. Injectable CPHs, introduced through minimally invasive interventions involving needles or catheters, can reduce the area of injury and inflammation resulting from surgery. Many injectable CPHs struggle with mechanical strength, conductivity, and complicated injection processes.^[^ ^23^ ^]^ One group synthesized an injectable polydopamine hydrogel with PEDOT:poly(styrene sulfonate) (PSS) as the conductive filler.^[^ ^24^ ^]^ This hydrogel features a nonconductive scaffold with a conducting polymer coating, which might present high impedance at the tissue‐electrode interface.^[^ ^25^ ^]^ Another group prepared an injectable conducting hydrogel from pure PEDOT:PSS with gelation times from 2 to 200 min.^[^ ^26^ ^]^ This method complicates surgeries because of the necessary tube mold. Without instant gelation, the liquid precursor can flow into surrounding areas, decreasing cross‐linking efficiency.^[^ ^27^ ^]^ Reports exist of crosslinking hydrogels made from various materials with gelation times from 42.4 to 252.1 s.^[^ ^28^ ^]^ Rapid gelation can match the mechanical stiffness of microenvironments and support the cavity.^[^ ^29^ ^]^ Currently, optic nerve repair, a complex part of the central nervous system, is rarely attempted. Scaffold materials are mainly applied to simpler peripheral nerve repair.^[^ ^30^, ^31^ ^]^ Traditional tissue engineering materials struggle to penetrate the optic nerve because of the CNS’ limited regeneration and Wallerian degeneration. Combining minimally invasive surgery with conductive hydrogels for optic nerve injuries continues to present a difficult problem.

We created a new, injectable CPH with PEDOT to restore function in injured optic nerves. We fabricated a PEDOT precursor solution by a simple crosslinking process with 4‐dodecylbenzenesulfonic acid (DBSA). Direct injection of this solution into a saline solution forms a wire‐like hydrogel, requiring no further treatment. This hydrogel formed instantly at the damaged optic nerve cavity to give structural support. The PEDOT hydrogel's elastic modulus matches that of nerve tissue. The good electroactivity of the PEDOT hydrogel benefits nerve signal transmission. The hydrogel's low impedance over 28 days indicates its ability to conduct high‐quality signals in vivo. Our experiments confirm that this hydrogel restores the electrophysiological function of optic nerves suffering from axonotmesis, shown by visual evoked potential and electroretinography tests. The PEDOT hydrogel also reduced retinal ganglion cells (RGCs) loss after axonotmesis.

## 2 Results and Discussion

**Figure 1:**
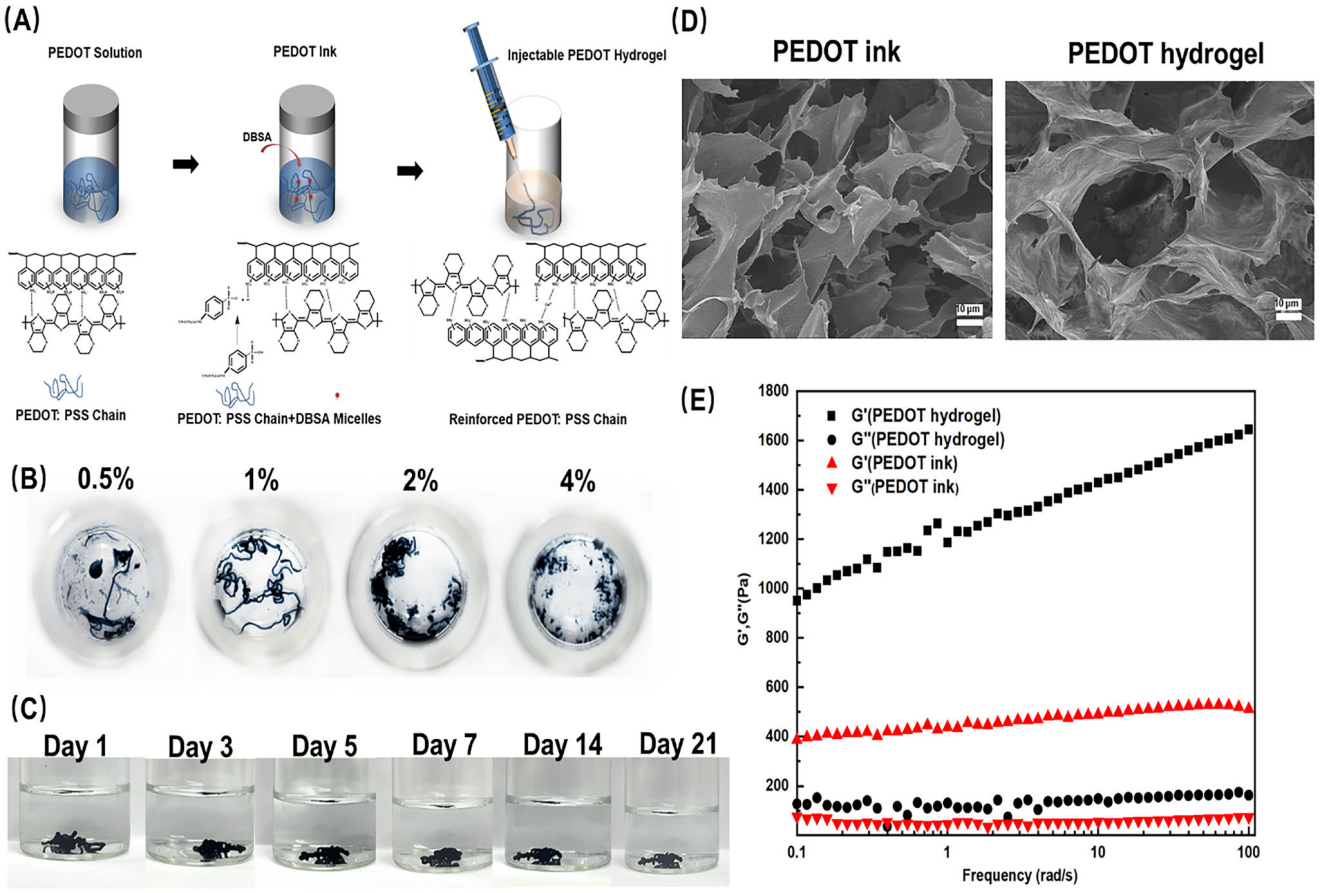
The preparation and rheological behavior of the injectable PEDOT hydrogel. A) Schematic procedures for fabricating the injectable PEDOT hydrogel. B) Digital images of the PEDOT hydrogel with varying DBSA concentrations. C) Photographs of the PEDOT hydrogel immersed in PBS for 21 days. D)Surface morphology of the PEDOT ink and the PEDOT hydrogel. E) Rheological behavior of the PEDOT ink and the PEDOT hydrogel.

In the PEDOT solution, the PSS^−^ chains encapsulate the hydrophobic PEDOT^+^ through electrostatic interactions. The formation of the injectable PEDOT hydrogel occurs in just two simple steps (**Figure** **1**A). In the first step, the PEDOT precursor solution is prepared by combining the PEDOT liquid with DBSA. DBSA provides H^+^ ions to protonate the PSS^−^ chains and facilitates the physical crosslinking of PEDOT^+^ chains through π–π stacking and hydrophobic interactions. In the second step, the resulting mixture serves as the “ink” and is injected directly into a saline solution, instantly forming a wire‐like hydrogel without the need for a mold. Bivalent ions, such as Ca^2+^, in the saline, interact electrostatically with the PSS^−^ chains, acting as ionic crosslinkers. This ionic crosslinking helps the hydrogel maintain its structure in saline, even after agitation, as opposed to when the ink is dispersed quickly in water without the presence of these salt ions (see Figure S1, Supporting Information).

The concentration of DBSA in the ink plays a critical role. Both the lower concentration (0.5 v/v%) and higher concentrations (2 v/v% and 4 v/v%) were found to be ineffective in forming a continuous wire‐like hydrogel in the saline (Figure 1B). The lower concentration of DBSA did not provide sufficient H^+^ ions to establish a robust network for the hydrogel injection, while higher concentrations led to the formation of excessive micellar structures bearing negatively charged sulfuric acid groups, resulting in clusters of hydrogel particles. We selected a 1 v/v% concentration of DBSA for all further experiments, unless otherwise specified. Figure 1C demonstrates the consistency of the PEDOT hydrogel after a 21‐day immersion in PBS, confirming the hydrogel's favorable long‐term stability.

The PEDOT ink and PEDOT hydrogel displayed distinct micro morphologies (Figure 1D). The ink exhibited a lamellar structure, where protonated PSS^−^ molecules compelled the PEDOT sheets to align in parallel through physical interactions. In contrast, the hydrogel presented a typical 3D cross‐sectional structure due to ion‐crosslinking. We confirmed the gel‐like behavior of the hydrogel by recording a frequency sweep measurement (see Figure 1E). The storage modulus G′ of the PEDOT hydrogel exceeded the loss modulus G″ across all frequency ranges, indicating the hydrogel's stability as an elastomer. The moduli exhibited slight increases within the 0.1–100 rad s^−1^ range, with G′ exceeding 1000 Pa and G″ reaching approximately 100 Pa. The hydrogel likely became more elastic (decreasing tan δ = G′′/ G′) as its structure stiffened dynamically.^[^ ^32^ ^]^ The G″ of the PEDOT hydrogel stayed at about 100 Pa, indicating that the PEDOT hydrogel retains its viscoelasticity throughout the entire frequency range tested. Because the PEDOT hydrogel maintained its form, all values remained above zero. Any deformation of the PEDOT hydrogel structure is reversed. The PEDOT hydrogel consistently showed greater G′ values than the PEDOT ink at similar loadings. The PEDOT hydrogel's elastic modulus (about 1000 Pa) matches the range of modulus values for nerve tissue (100‐3000 Pa).^[^ ^33^, ^34^ ^]^ This data confirms that the newly prepared PEDOT hydrogel is well‐suited for biological implantation.

**Figure 2:**
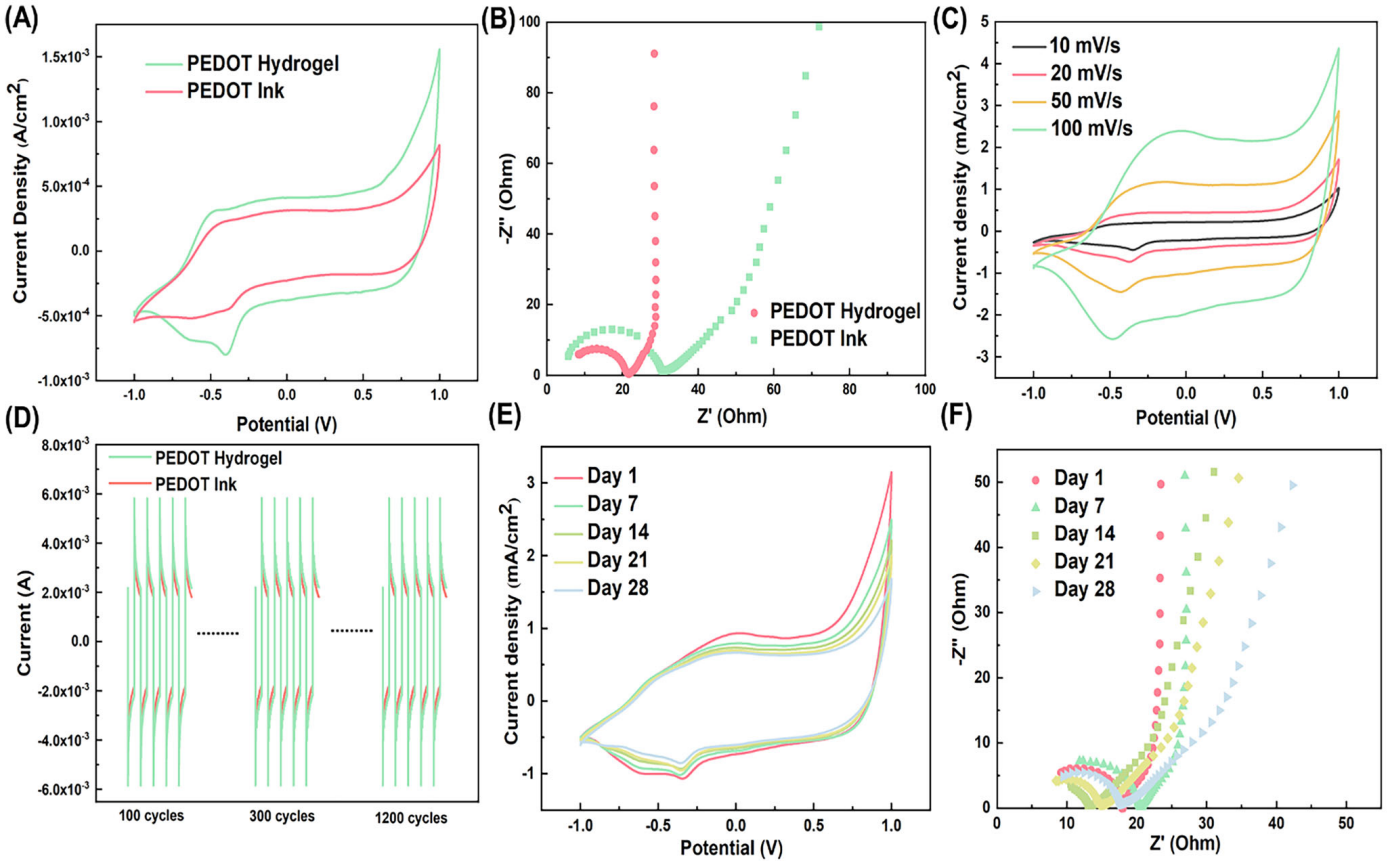
Electrochemical properties of the PEDOT hydrogel. A) Cyclic voltammograms (CVs) of the PEDOT hydrogel and PEDOT ink in PBS at a scan rate of 20 mV s^−1^. B) Nyquist plot of the PEDOT hydrogel and PEDOT ink in PBS. C) CVs of the PEDOT hydrogel at different scan rates in PBS; D) Double potential step chronoamperometry of the PEDOT hydrogel and the PEDOT ink in a PBS solution with 1200 cycles. The potential was stepped from +0.1 to 0 and back to ‐0.1 V. E) CVs of the PEDOT hydrogel in PBS at day 1, day 7, day 14, day 21, and day 28, scan rate = 20 mV s^−1^. F) Nyquist plot of the PEDOT hydrogel in PBS at day 1, day 7, day 14, day 21, and day 28.

Cyclic voltammograms (CVs) represent a simple and rapid technique for assessing capacitance and Faradaic components at the electrode‐solution interface.^[^ ^35^, ^36^ ^]^ In comparison to the PEDOT hydrogel, the PEDOT ink exhibited lower current densities for both oxidation and reduction (**Figure** **2**A). The porous structure of the PEDOT hydrogel accounts for its greater current response, promoting swift electron and ion movement.^[^ ^37^ ^]^ Both electrodes displayed minimal bulk resistance, approximately 5 Ω (Figure 2B), confirming their excellent electrical properties suitable for nerve conduction. The PEDOT hydrogel exhibited a much smaller charge transfer resistance, as evidenced by the semicircle in the Nyquist plot, indicating faster electrochemical kinetics within its 3D continuous nanostructured framework. The nearly vertical curve shape at lower frequencies signified ideal capacitive behavior of the PEDOT hydrogel.^[^ ^38^ ^]^ The shape of CV curves remained consistent with increasing scan rates from 10 to 100 mV s^−1^, while current density gradually increased with higher voltage scan rates (Figure 2C). The Randles–Ševčík equation, which describes the relationship between peak current and scan rate in CVs can be applied based on the analysis of Figure 2C:

(1) $$i_{p}=kv^{1/2}c$$

where c is constant. A value of k = 0.5 reflects an ideal Faradaic insertion/extraction, while k = 1 represents a capacitive response. The k value of the PEDOT hydrogel was 0.775 obtained from Figure S2 (Supporting Information), suggesting a mixed mechanism during charge storage. We evaluated the cyclic stability of the PEDOT hydrogel as a conductor using chronoamperometry, as shown in Figure 2D. Tested at a step potential of ±1 V, it demonstrated high cyclic stability,^[^ ^39^ ^]^ with peak current density and current curve shape remaining unchanged after 1200 cycles. The electrochemical performance of the PEDOT hydrogel was consistently maintained over an extended period (Figure 2E,F), making it suitable for implantable applications as a conductor. The CV curves were nearly overlapping during this period (see Figure 2E), and the nearly vertical shape at lower frequencies remained consistent, indicating that the capacitive property was preserved over time (Figure 2F). The prepared PEDOT hydrogel had a high impedance (≈18.0 Ω at 1 kHz). We considered the impedance data at 1 kHz because most neuron communication happens between 300 Hz and 1 kHz.^[^ ^40^ ^]^ The PEDOT hydrogel's impedance is far lower than that of other PEDOT hydrogels used for nerve tissue engineering, as reported in other works.^[^ ^28^, ^41^, ^42^ ^]^ The impedance of the PEDOT hydrogel in this study stayed at 17.3 (±2.7) Ω after 28 days, indicating its high long‐term stability. The low impedance helps to record high‐quality signals when applying the hydrogel in vivo. Electrochemical tests confirmed the prepared PEDOT hydrogel's good electroactivity and high ionic conductivity, which can promote nerve signal transmission.

**Figure 3:**
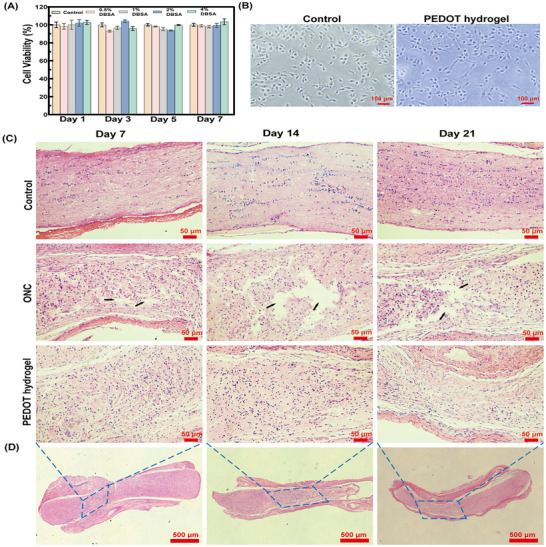
Biocompatibility of the PEDOT hydrogel. A) In vitro cytotoxicity of the PEDOT hydrogel with varying concentrations of DBSA (0.5%, 1%, 2%, and 4%) at Day 1, Day 3, Day 5, and Day 7. B) Microscopy image of R28 cells cultured on the PEDOT hydrogel after 24 h. C) Hematoxylin and eosin (H&E) staining of the optic nerve in the control, the optic nerve crush (ONC) group, and the PEDOT hydrogel group, harvested at Day 7, Day 14, and Day 21. The arrows in the ONC group point to the absence of axons, forming a cavity. D) H&E staining at a lower magnification. The blue area indicates the presence of the PEDOT hydrogel.

The biocompatibility of the PEDOT hydrogel with varying proportions of DBSA was assessed using PC12 cells, a commonly used cell type in nerve research. Cell Counting Kit‐8 (CCK8) analysis revealed similar proliferative activity in both the control and experimental groups on days 1, 3, 5, and 7 (**Figure** **3**A). Additionally, retinal precursor cells (R28) cultured on the PEDOT hydrogel demonstrated effective attachment, as shown in the representative microscopy images on day 3 (Figure 3B). The SEM image in Figure S3 (Supporting Information) displayed R28 cells spreading extensively within the PEDOT hydrogel scaffold's porous architecture. Hematoxylin and eosin (H&E) staining (Figure 3C) supported these findings, with optic nerve crush (ONC) groups showing minimal signs of inflammatory response or immune rejection (black arrows) and no necrotic tissue near the PEDOT hydrogel. These observations indicate that the PEDOT hydrogel is biocompatible with optic nerve tissue. Figure 3D displays the complete optic nerve structure after H&E staining. The blue region marks the location of the PEDOT hydrogel. The hydrogel remained unchanged 21 days after in vivo injection. Figure S4 (Supporting Information) shows the PEDOT hydrogel's capacity to fill the damaged area effectively, offering structural support.

**Figure 4:**
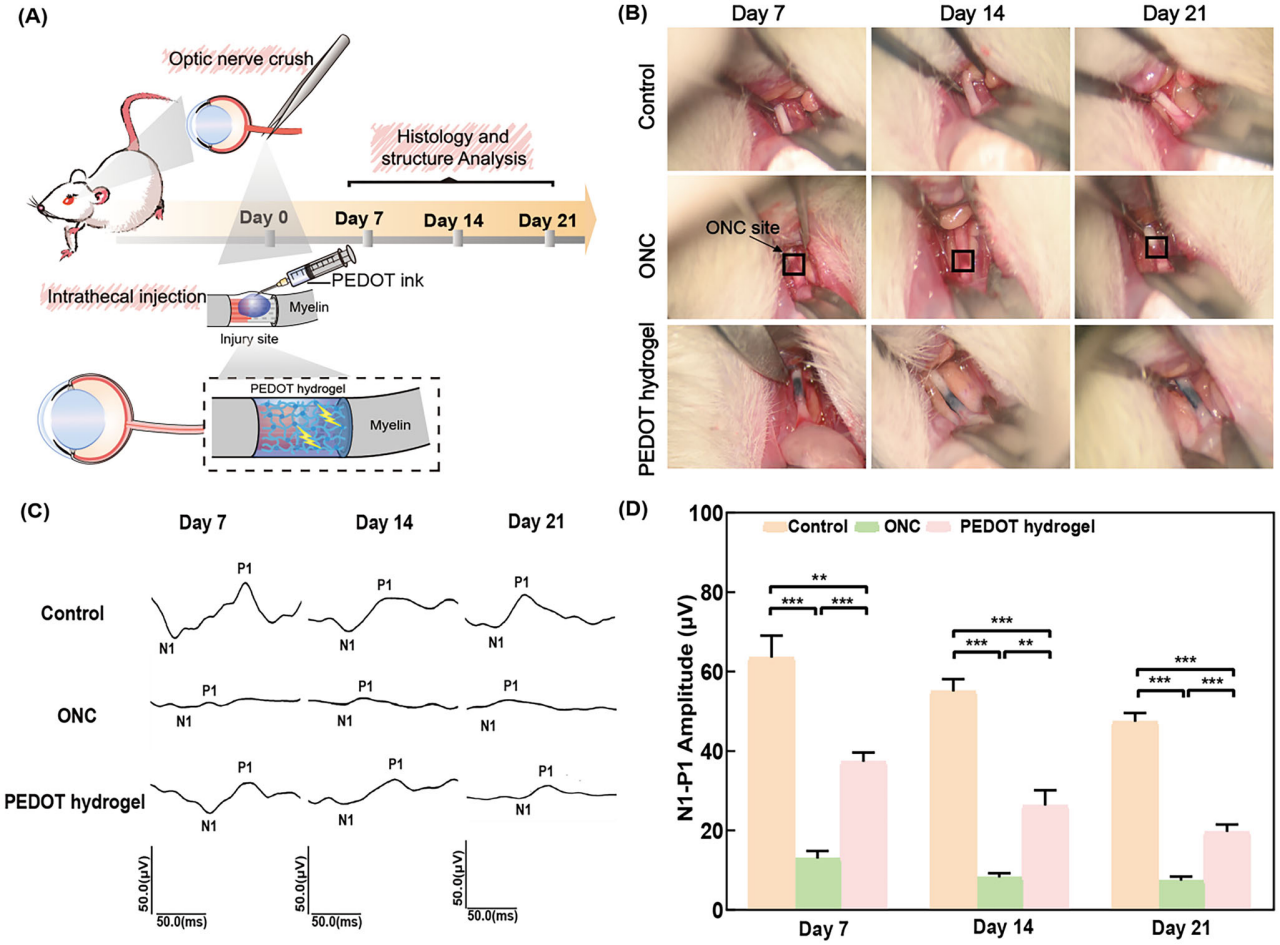
Surgical implantation, operation, and replacement for the injured optic nerve in a rat model. A) Schematic illustration of the ONC group, and injection of the PEDOT hydrogel group. B) Photographs of optic nerves in rats from the control group, ONC group, and PEDOT hydrogel group on day 7, day 14, and day 21. C) Representative flash visual evoked potentials tracings from the control group, ONC group, and PEDOT hydrogel group at day 7, day 14, and day 21. D) Statistical analysis of the N1‐P1 amplitude in each group. The data are presented as mean ± SEM (n ≥ 6). ^*^ *p* < 0.05, ^***^ *p* < 0.001.

To assess the therapeutic efficacy of the PEDOT hydrogel in vivo, PEDOT ink was injected into the injury site of the myelinated optic nerve, creating a highly conductive bridge across the nerve gap (**Figure** **4**A). The PEDOT hydrogel was used to replace the damaged nerves in vivo and evaluated three weeks postoperatively (Figure 4B). The exposed nerve length was approximately 3 mm, and the filled gap measured around 1 mm. Notably, the ONC group exhibited septic collections throughout the observation period in the ONC site. In contrast, the PEDOT hydrogel group showed that the myelinated optic nerve gap generally maintained lumen and wall integrity in all tested animals, with alleviated symptoms. They didn't display the significant tissue adhesions and exudation of turbid tissue fluid around the optic nerve.

Flash visual evoked potentials (FVEP) is a neurophysiological method used to assess electrical activity induced by flash stimulation of the retina. This method reflects the comprehensive conduction of visual information along the optic pathway from the retina to the visual cortex.^[^ ^43^ ^]^ The amplitude of the N1‐P1 wavelet serves as an indicator of retinal response to light stimulation and axonal transmission of visual information. As depicted in Figure 4C, when the nerve was entirely damaged by ONC, bioelectric signals failed to transmit. However, after hydrogel injection (PEDOT hydrogel group), significant FVEP responses were observed compared to the ONC group. FVEP assessments indicated that the N1‐P1 amplitudes in the control, ONC, and PEDOT hydrogel groups were 63.5, 12.9, and 37.3 µV, respectively (Figure 4D) at day 7. While a disparity existed between the intact optic nerve and the PEDOT hydrogel group due to the unavoidable injury following the crush surgery, the N1‐P1 amplitude was 2.9‐fold higher in the PEDOT hydrogel group than that in the ONC group (^***^ *p * < 0.001). This trend remained consistent at day 14 and day 21, with the PEDOT hydrogel group showing a 3.2‐fold increase over the ONC group at day 14 and a 2.7‐fold increase at day 21. FVEP measurements demonstrated that the PEDOT hydrogel significantly improved visual function following ONC.

**Figure 5:**
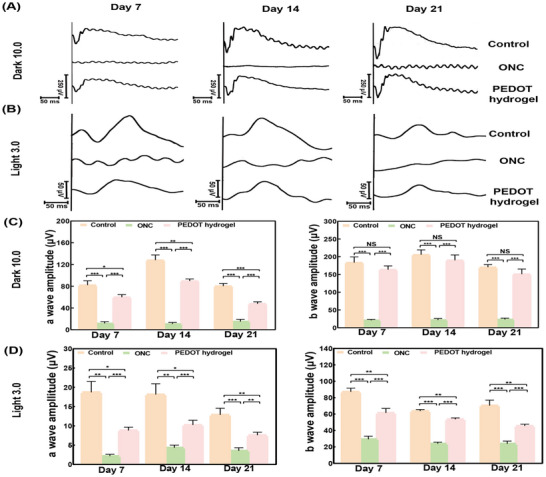
A representative electroretinography (ERG) results. A) ERG waves adapted at Dark 10.0, and B) at Light 3.0. Statistical Analysis of C) Wave amplitudes at dark 10.0, D) Wave amplitudes at Light 3.0. The data are presented Mean ± SEM (n ≥ 6). *p < 0.05, **p < 0.01, ***p < 0.001.

The findings were consistent with the patterns observed in the electroretinography (ERG) and the accompanying statistical analysis. Previous research has shown a decrease in the ERG waveform following modeled optic nerve damage.^[^ ^44^ ^]^ The ERG evaluations were conducted under conditions of both the dark adaptation (10.0) and light adaptation (3.0). As depicted in **Figure** **5**A,B, the damaged photopic and scotopic a‐wave and b‐wave responses in the ONC group exhibited improvement following the application of the PEDOT hydrogel. Specifically, after dark adaptation, the amplitude of the a‐wave decreased by more than 85%, and the b‐wave's amplitude declined by over 90% in the ONC group, when compared to the control (Figure 5C). Similarly, during the light adaptation, the a‐wave's amplitude reduced by over 80%, while the b‐wave's amplitude decreased by more than 70% in the ONC group compared to the control (Figure 5D). These findings indicate the extent of severe damage to the optic nerve and retina, leading to vision problems. However, treatment with the PEDOT hydrogel significantly restored visual function. Throughout the observation period, both a‐wave and b‐wave amplitudes in the PEDOT‐treated groups exceeded those in the ONC group, under both dark and light conditions. In fact, most amplitude values in the PEDOT groups were comparable to those in the control group. This supports that the visual deficits resulting from ONC can effectively rehabilitated through the incorporation of the PEDOT hydrogel.

The regrowth potential was evaluated through the expression of growth‐associated protein GAP‐43, which plays an important role in establishing neural circuitry. After injury, the ONC group and the PEDOT hydrogel group exhibited limited expression compared to controls (Figure S5, Supporting Information). This indicates that treatment with the PEDOT hydrogel didn't promote the regrowth of optic nerve. Healthy adult RGCs typically express Thy‐1, a retinal cell surface glycoprotein, on their cell bodies.^[^ ^45^ ^]^ Thy‐1 is vital for the stability and synaptic plasticity of neural networks. Figure S6 (Supporting Information) shows that Thy‐1 expression decreased sharply in the ONC group. A white frame marks the trailing end of the injury. Applying the PEDOT hydrogel caused Thy‐1 labeled axons to adhere tightly to the hydrogel, preventing degenerative contraction of the axon's distal end. This observation suggests the PEDOT hydrogel acts as a bridge, promoting bioelectrical signal transmission.

**Figure 6:**
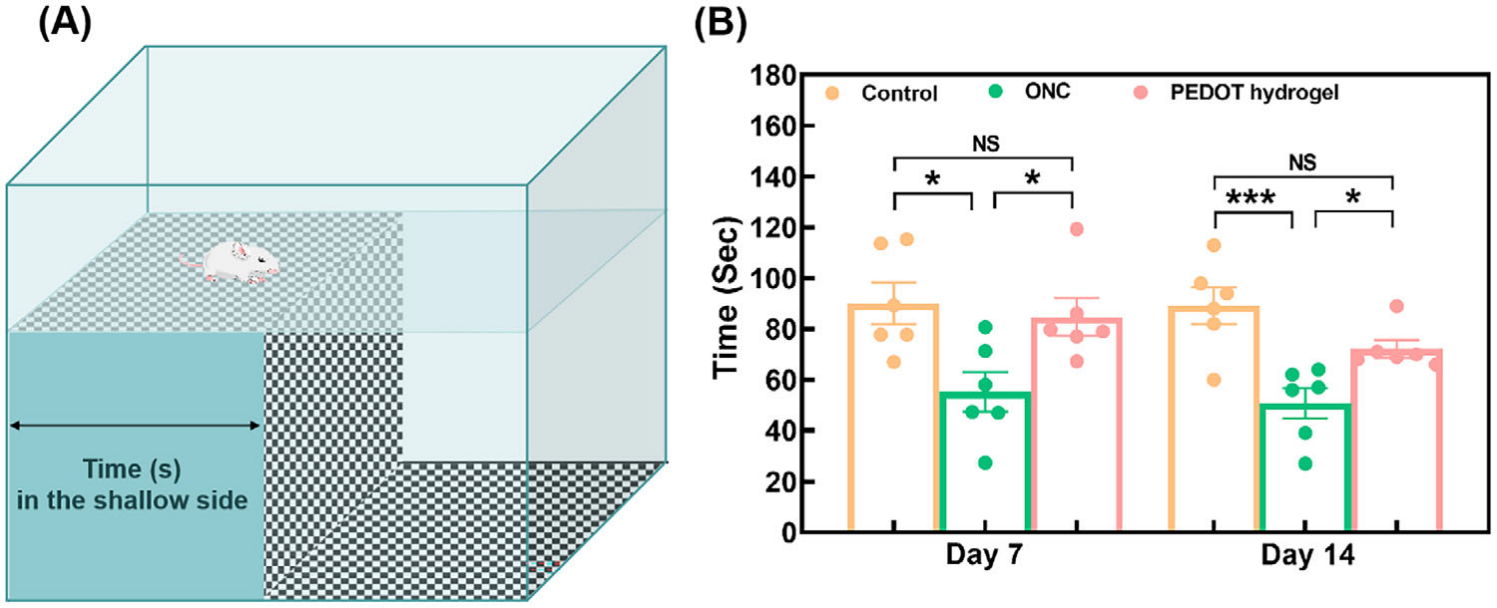
The results of the visual cliff test. A) A schematic diagram of the visual cliff test. B) Statistics of the visual cliff test data on Day 7 and Day 14. The data that is shown has a mean ± SEM (n = 6). ^*^ *p* < 0.05, ^***^ *p* < 0.001.

The visual cliff test assessed visual function and spatial perception (**Figure** **6**). Rats in the PEDOT hydrogel group stayed at the shallow end 94.0% at day 7 and 80.9% at day 14 of the time during the entire test, close to that of the control group (which is 100%). This value is much higher than that in the ONC group, which is 58.0% at day 7 and 57% at day 14.

**Figure 7:**
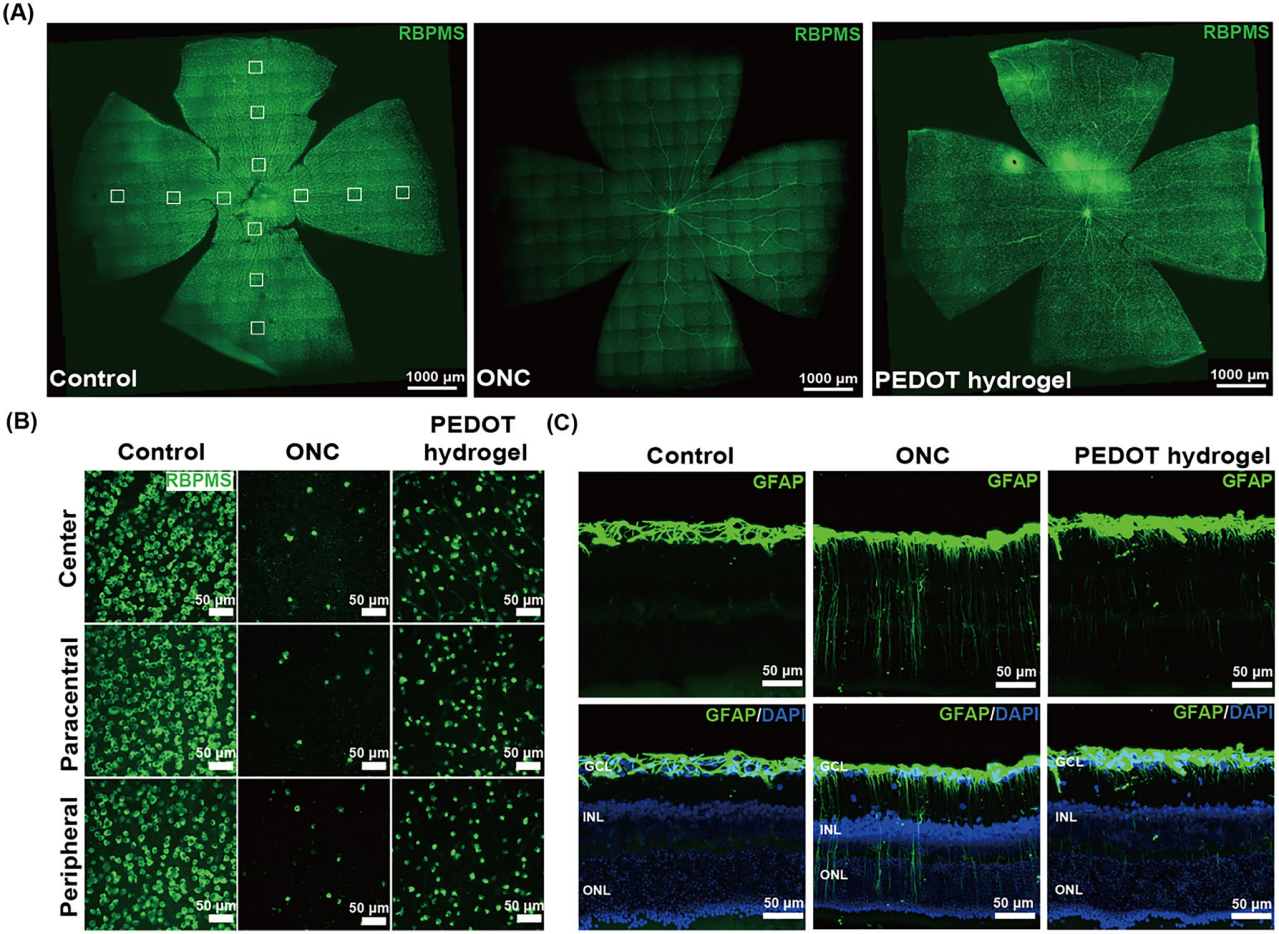
RGC Survival in the Control, the ONC, and the PEDOT hydrogel group. A) Density distribution of retinal ganglion cells (RGCs) in whole‐mount retinas in different groups, Scale bar = 1000 µm. B) Panels B (20× magnification) show magnified micrographs of center, paracentral, and peripheral RGCs in different groups. Scale bar = 50 µm. C) Immunofluorescent labeling for GFAP performed on retinal sections at Day 14. Inner nuclear layer (INL), outer nuclear layer (ONL), and DAPI nuclear counterstain (blue label) was demonstrated. D) Stainning images of c‐fos on the optic nerve.

ONC injury leads to apoptosis in RGCs. In around 90% of RGCs they undergo apoptosis within 14 days of ONC.^[^ ^46^ ^]^ We assessed the protective effect of the PEDOT hydrogel by quantitatively determining the number of RGCs in the retina following ONC injury. Whole‐mount retinas were used, and the specific marker RBPMS was employed to label RGCs. RGCs were found to be evenly distributed in the retinas throughout (**Figure** **7**A) and in different retinal regions (Figure 7B).

In the 14‐day ONC groups, there was a decline in the total number of RGCs, which dropped by a staggering 95% (111 ± 5 cells mm^−2^). However, the introduction of PEDOT hydrogel proved to be a safeguard for RGCs, resulting in a fourfold increase in the count of surviving RGCs (517 ± 36 cells mm^−2^) when compared to the ONC group. It's important to note that though the relative RGC viability rate in the PEDOT hydrogel group remains below that of the control (Figure S7, Supporting Information), the enhancement in RGC protection observed in this study represents an advancement in restoration of the optic nerve. This protective effect was consistent across different retinal regions (Figure S8, Supporting Information), indicating a significant preservation of RGCs and their electrophysiological function following injury, provided by the PEDOT hydrogel.

The Müller cell is a primary glia cell throughout the whole retina. Müller cell has been considered as major factor for the RGC restoration at the initial stage of the retinal injury. They have been recognized both as facilitators of RGC restoration and as potential contributors to RGC injury when their proliferation becomes excessive.^[^ ^47^ ^]^ The interaction between Müller cells and RGCs is important in shaping the retinal microenvironment and supporting RGC recovery. In Figure 7C and Figure S9 (Supporting Information), we illustrated the expression of the glial fibrillary acidic protein (GFAP), a protein associated with Müller cells.^[^ ^48^ ^]^ Notably, the ONC group at Day 14 exhibited an overexpression of GFAP, indicating excessive Müller cell proliferation when compared to the control (Figure S10, Supporting Information). However, with the application of PEDOT hydrogel, the expression of GFAP was attenuated. This, in turn, promotes the restorative function of Müller cells in aiding RGC restoration, as depicted in Figure 7A,B.

Further investigation was carried out to examine the immune functions associated with retinal factors such as microglia. Glial activation, which triggers the release of inflammatory cytokines, is a characteristic of retinal injury caused by ONC and is implicated in the secondary degeneration of RGC soma.^[^ ^49^ ^]^ ONC triggers neuronal apoptosis in the RGCs, which activates resident retinal microglia and recruitment of infiltrating macrophages into the retinae and proximal nerve stump.^[^ ^47^ ^]^ In adult female rats, an increased number of microglia was detected in the retina as early as 2 days after ONC and accompanied by a rise in neurotoxic pro‐inflammatory cytokines in the retinae and proximal nerve stump. This resulted in widespread neuronal loss and axon degeneration after ONC. Moreover, ONC triggers not only the activation of resident retinal microglia, but also other microglia in the retinae, including astrocytes and Müller cells.^[^ ^50^ ^]^ As illustrated in Figures S11 and S12 (Supporting Information), there was an increase in the activation of microglial cells in the ONC group when compared to control group. In the outer plexiform layer of the retina, the total number of microglial cells was amounted to be 126 ± 4 cells mm^−2^ in the ONC group (Figure S13, Supporting Information). This number was reduced by 50% to 63 ± 2 cells mm^−2^ in the PEDOT hydrogel group, indicating the effective suppression of microglial activity with the incorporation of PEDOT hydrogel. A similar outcome was observed in the ganglion cell layer of the retina (Figures S14 and S15, Supporting Information). Following ONC, the application of PEDOT hydrogel resulted in a 36.5% reduction in the total number of microglia in the ganglion cell layer of the retina (Figure S16, Supporting Information). These pathological findings suggest that the administration of PEDOT hydrogel at the injury site can alleviate neuroinflammation in the retina and serve as a preventive measure against RGC loss. Neural transmission operates through electrochemistry.^[^ ^51^ ^]^ Transmembrane potentials drive ionic movement into and out of neurons, and this movement generates nerve impulses. Various ionic channels, such as calcium, sodium, and potassium channels, mediate this process.^[^ ^52^ ^]^ Injury to RGC axons (which make up the optic nerve) disrupts ionic transport and signal transmission between the axons and cell bodies. The integration of the PEDOT hydrogel presents alternate routes for ionic transport and neural signal transmission. The protein C‐fos is a downstream factor of calcium.^[^ ^53^ ^]^ Figure S17 (Supporting Information) displays that treatment with the PEDOT hydrogel in the ONC group increased c‐fos expression. This observation confirms that integration of the PEDOT hydrogel stimulates the calcium‐dependent signaling pathway.

## 3 Conclusion

Our work developed an innovative injectable CPH based on PEDOT. This hydrogel can rapidly gel at the site of axonotmesis in the optic nerve, achieving small trauma treatment to fit irregular defective tissue. The conductive hydrogel, featuring excellent electrochemical properties, mechanical characteristics akin to nerve tissue, and outstanding biocompatibility, has demonstrated its capacity to effectively replace missing optic nerve tissue in vivo, thereby restoring electrophysiological function and facilitating the transmission of bioelectrical signals. Not only does our hydrogel help preserve RGCs, but it also holds promise in alleviating neuroinflammation by suppressing microglial activity and gliosis. This hydrogel remains stable in the biological body for a long time, providing it with a space for survival and nutrient storage. We envision that this injectable hydrogel has the potential for custom graft applications in cases of optic nerve axonotmesis within the realm of tissue engineering. This work also provides a biocompatible electroactive matrix with persistently stimulating regenerative bands and targeting the release of growth factors, which could serve as the primary direction for nerve graft industrialization.

## 4 Experimental Section

### Materials

The PEDOT: PSS pellets were sourced from Agfa Company, and DBSA was obtained from Sigma Aldrich.

### Fabrication of the Injectable PEDOT/DBSA Hydrogels

Various concentrations of PEDOT solutions (0.1, 0.5, 1, 1.5, and 2 w/v% in H_2_O) were prepared and mixed with DBSA (0.5, 1, 2, and 4 v/v%) under consistent magnetic stirring. The resulting slightly cross‐linked solution could be easily withdrawn using a syringe and then injected into PBS for the second cross‐linking step to form the final hydrogel. Unless specified otherwise, the hydrogel used in this work was composed of PEDOT (1.5 w/v%) and DBSA (1 v/v%) to ensure suitable mechanical properties.

### Characterization of the PEDOT Hydrogel and PEDOT Ink

The surface morphology of freeze‐dried hydrogels of the PEDOT hydrogel and PEDOT ink was investigated by field emission scanning electron microscopy (HITACHI, SU8010). Rheological experiments were conducted on a TA AR2000 rheometer with a parallel plate configuration (40 mm diameter) at a constant strain (0.1%) while the frequency was swept from 0.1 to 100 rad s^−1^. CV tests were performed with an electrochemical workstation (CHI 760D). The electrode was created by coating the testing materials on carbon cloth.

### In Vitro Cytotoxicity

PC12 cells (Wuhan Procell Life Technology, Wuhan, China) were cultivated in RPMI 1640 Medium (Gibco, Waltham, USA) supplemented with 10% heat‐inactivated fetal bovine serum (FBS, Gibco) and 0.2% gentamicin solution (50 mg/mL, Gibco). Retinal precursor (R28) cells (Biobw biotechnology, Beijing, China) were cultivated in DMEM high glucose Medium (Gibco, Waltham, USA) supplemented with 10% heat‐inactivated fetal bovine serum (FBS, Gibco) and 0.2% gentamicin solution (50 mg mL^−1^, Gibco). The cells were then incubated in a humid environment at 37 °C with 5% CO_2_. The PEDOT hydrogel was sterilized beforehand. R28 cells were directly seeded onto the PEDOT hydrogel and incubated for 24 h, after which pictures were taken with a forward microscope. The biocompatibility of PEDOT hydrogel was assessed using a CCK‐8(Dojindo, Kumamoto, Japan) assay. PEDOT hydrogel at various concentrations was added to the cell wells and co‐cultured with PC12 cells for 24 to 168 h, followed by washing the cells with DPBS. Then, 10 µL of CCK‐8 solution were added to each well. The culture plate was incubated for 0.5 to 2 h, and the absorbance of the culture media was measured using a microplate reader (SpectraMax M5, Molecular Devices, San Jose, CA, USA) at 450 nm.

### Surgical Procedure

Rats were anesthetized via intraperitoneal injection of a 1% sodium pentobarbital solution (25 mg kg^−1^). A 2 mm incision was made from the bulbar conjunctiva of the lateral canthus to bluntly separate the extraocular muscle and intraorbital fat. The ONC group clamped the optic nerve for 5 s at 2 and 3 mm behind the bulb, and then punctured on the myelin sheath with a micro syringe needle, finally squeezed out the optic nerve axons at the clamped site using toothless forceps. In the PEDOT group, the operation was performed as above, a Hamilton syringe needle (30 G) was inserted posterior to the axon deletion site, and ≈1.5 µL of PEDOT hydrogel was slowly injected. After the procedure, the fundus was promptly examined ophthalmologically to rule out any rats with perfusion malfunction because of the procedure. The two eyes of the rats were different treatment groups in the following experiments, except for the visual cliff test.

### Ethical Conduct of Research

The animal research protocols were approved by the Laboratory Animal Ethics Committee of Wenzhou Medical University (Approval number: wydw2022‐0144), and all experiments adhered to the principles outlined in the Declaration of Helsinki.

### Electroretinograms

ERG and VEP in rats were conducted with the RETI‐port ERG system (Ganzfeld Q450 SC; Roland Consult, Wiesbaden, Germany). SD rats were dark‐adapted in advance for 6 h. Rats were anesthetized by intraperitoneal injection of sodium pentobarbital at 50 mg kg^−1^. The eyes were anesthetized by surface anesthesia with proparacaine hydrochloride eye drops. Pupils were dilated with compound tropicamide eye drops. Ring shaped gold recording electrodes were placed on the cornea of both eyes. A pair of reference needle electrodes made of stainless steel were placed subcutaneously behind the ears and a ground electrode was placed subcutaneously by the tail. The ERG parameters for photopic responses included stimulation intensities at 0.48 log candela (cd) s m^−2^ (light 3.0). The ERG parameters for scotopic responses featured stimulation intensities at 0.98 log cd·s m^−2^ (dark 10.0).

### Visual Evoked Potential

Rats were anesthetized by intraperitoneal injection of sodium pentobarbital at 50 mg kg^−1^. The recording electrode was inserted subcutaneously from the position of the midpoint of the line connecting the roots of the two ears, and the tip of the needle was positioned exactly corresponding to the midline of the mouse optic cortex. The reference potential was inserted subcutaneously in the cheek and the grounding electrode was inserted subcutaneously in the tail root. One eye was being tested while the other eye was covered. The VEP parameters included a filter frequency of the signal collector from 1.0 to 100.0 Hz, flash stimulus at a rate of 1.4 Hz, a test average of 100 sweeps, and a stimulus strength of 3 cd·s·m^−2^.

### Visual Cliff Test

The visual cliff platform consists of two 50 × 25 cm transparent plates, divided into shallow and deep ends. A 1.8 × 1.8 cm black and white checkerboard pattern was placed directly below the transparent plates at the shallow end. At the deep end, the black and white checkerboard pattern was placed 35 cm below the transparent plates. At the beginning of the experiment, the rats were placed at the shallow end, and their behavior was recorded with a video camera for 2 min. Each rat was tested thre times with a 5‐min interval for rest. The platform was cleaned with 75% alcohol after each test. Statistical analysis was performed concurrently at the shallow end.

### Immunofluorescence Staining of Retinal Frozen Sections and Whole‐Mount Retina

Enucleated eyes or whole retinas from Sprague‐Dawley rats were fixed with 4% paraformaldehyde (PFA) for 30 min. Enucleated eyes were dehydrated overnight at 4 °C in 30% sucrose solution and then embedded in optimal cutting‐temperature tissue embedding agents. The eyes were sectioned to a thickness of 15 µm. Sections and retinas were permeabilized with 0.2% Triton X‐100 and blocked with 5% BSA. Sections and retinas were incubated overnight at 4 °C with primary antibodies against anti‐GFAP (Abcam, ab33922, 1:500), anti‐Iba1 (Wako, 019–19741, 1:500), anti‐RBPMS (Proteintech, 15187‐1‐AP, 1:500) and anti‐c‐fos (Abcam, ab302667, 1:250). Following three rinses with PBS for 5 min each, an Alexa Fluor‐488 conjugated secondary antibody (Abcam, ab150077, 1:500) and a FITC conjugated secondary antibody (HUABIO, HA1003, 1:500) was applied and incubated for 2 h at room temperature. The final samples were covered and slipped with a DAPI‐containing anti‐fluorescence quencher, and the immunofluorescence staining images were observed and photographed by confocal scanning laser microscopy (Zeiss LSM 900). Immuno‐positive cells were counted under fluorescent illumination (20×) and averaged to estimate cell numbers per square millimeter (n = 4, mean ± S.E.M.).

### Western Blotting

Retina samples were isolated from different groups of rats at various time points. Fresh‐frozen retina samples were supplemented with 150 µL of tissue lysis buffer (1% PMSF and 99% RIPA), and the concentration was measured using a BCA assay. The protein was loaded and separated on a 12% SDS‐PAGE gel and then transferred to polyvinylidene fluoride membranes. The membrane was blocked in 10% nonfat milk in TBST for 1 h. It was incubated overnight with anti‐GFAP antibodies (Abcam, ab33922, 1:1000) and anti‐GAPDH (Abcam, ab9485, 1:2000). The membrane was then incubated with goat anti‐rabbit (HRP) (Abcam, ab205718, 1:5000). The washed membranes were visualized with an ECL kit.

### Statistical Analyses

The data were presented as the means ± SEM, and statistical significance was assessed using analysis of variance (ANOVA). GraphPad Prism 7.0 software was used for statistical analysis. Statistical significance was indicated as ^*^ *p * < 0.05.

## Conflict of Interest

The authors declare no conflict of interest.

## Supporting information



Supporting Information

## Data Availability

The data that support the findings of this study are available in the supplementary material of this article.
