# Supplementary material for: A Facile Strategy to Restore the Optic Nerve Functionality Using an Injectable Conducting Hydrogel
Source: Adv Sci (Weinh). 2025 Apr 17;12(21):2415601. doi: 10.1002/advs.202415601 (PMC12140289; doi:10.1002/advs.202415601)

Supplemental Information for

**A Facile Strategy to Restore the Optic Nerve Functionality using An Injectable Conducting Hydrogel**

*Changchun Yu,^1,2^ Yandi Zhou,^1,2^ Shuang Yao,^1,2,3,4^ Ziyi Wang,^1,2^ Sihao Ye,^1,2,3^Rubing Qi,^1,2^ Hai Hu,^1,2^ Keke Liu,^1,2^ Yabo Wu,^5^Tom Lawson,^6^ Lu Yan,^1,2,*^ Yong Liu^1,2,*^ and Wencan Wu^1,2,3,4,*^*

^1^School of Ophthalmology and Optometry, School of Biomedical Engineering, Wenzhou Medical University, 270 Xuanyuanxi Road, Wenzhou, Zhejiang 325027, China

^2^National Engineering Research Centre of Ophthalmology and Optometry, Eye Hospital, Wenzhou Medical University, Wenzhou, Zhejiang 325027, China

^3^Zhejiang Key Laboratory of Key Technologies for Visual Pathway Reconstruction, Wenzhou Medical University, Wenzhou, Zhejiang 325000, China

^4^Oujiang Laboratory (Zhejiang Laboratory for Regenerative Medicine Vision and Brain Health), Wenzhou, Zhejiang 325000, China

^5^School of Biomedical Engineering and Imaging Sciences,

King’s College London

London, WC2R2LS, United Kingdom

^6^School of Chemical Engineering, University of New South Wales NSW  2052, Australia

Corresponding author emails^*^: yanlu2107@wmu.edu.cn; yongliu@wmu.edu.cn; and wuwencan@wmu.edu.cn

**Figure S1.** Digital images of PEDOT ink injected into PBS solution and water. Left: Fresh samples. Right: Samples after shaking.


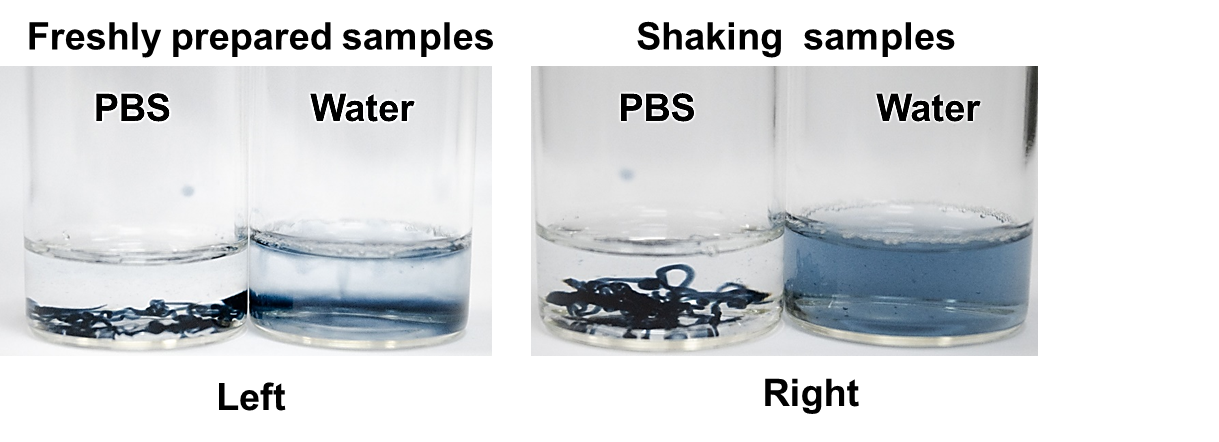


**Figure S2.** Cathodic peak currents versus the square root of scan rate. Data obtained from **Figure 2C**.


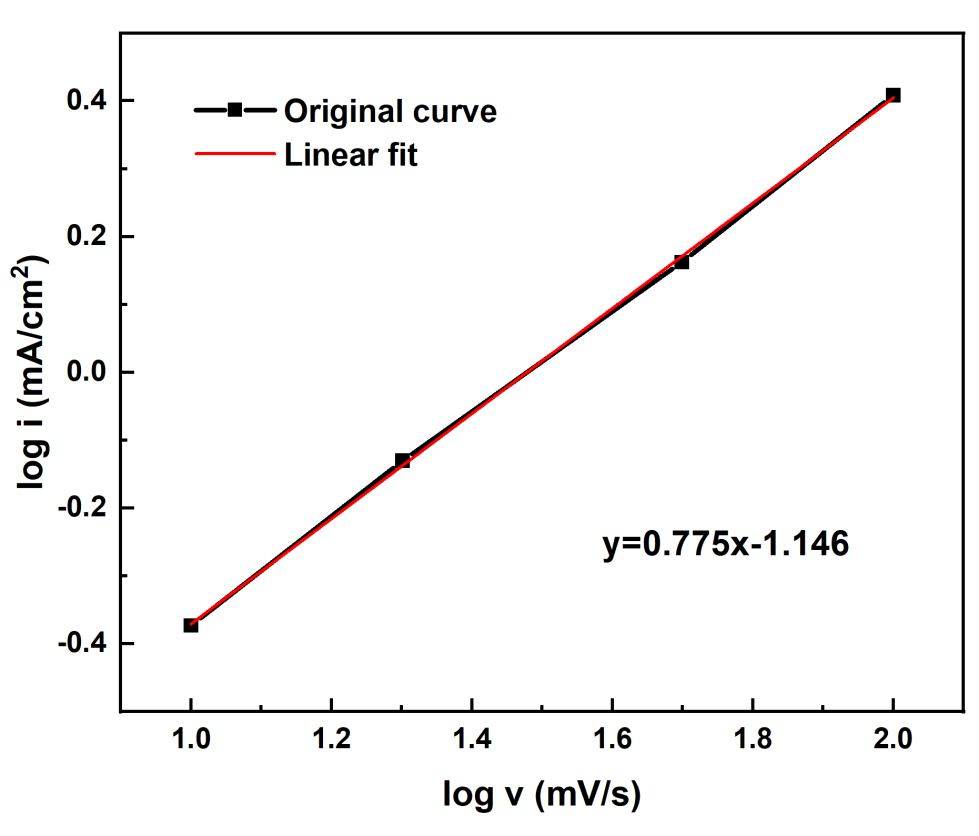


**Figure S3.** SEM image of R28 cells (indicated by the white arrows) grown within the PEDOT hydrogel scaffold's porous architecture.

**
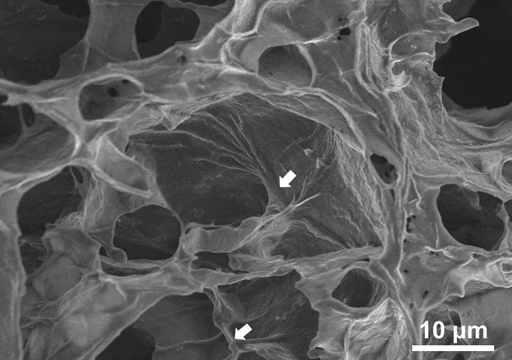
**

**Figure S4.** SEM images of the hydrogel in the optic nerve (A) and the intact optic nerve (B) after 21 days.

**
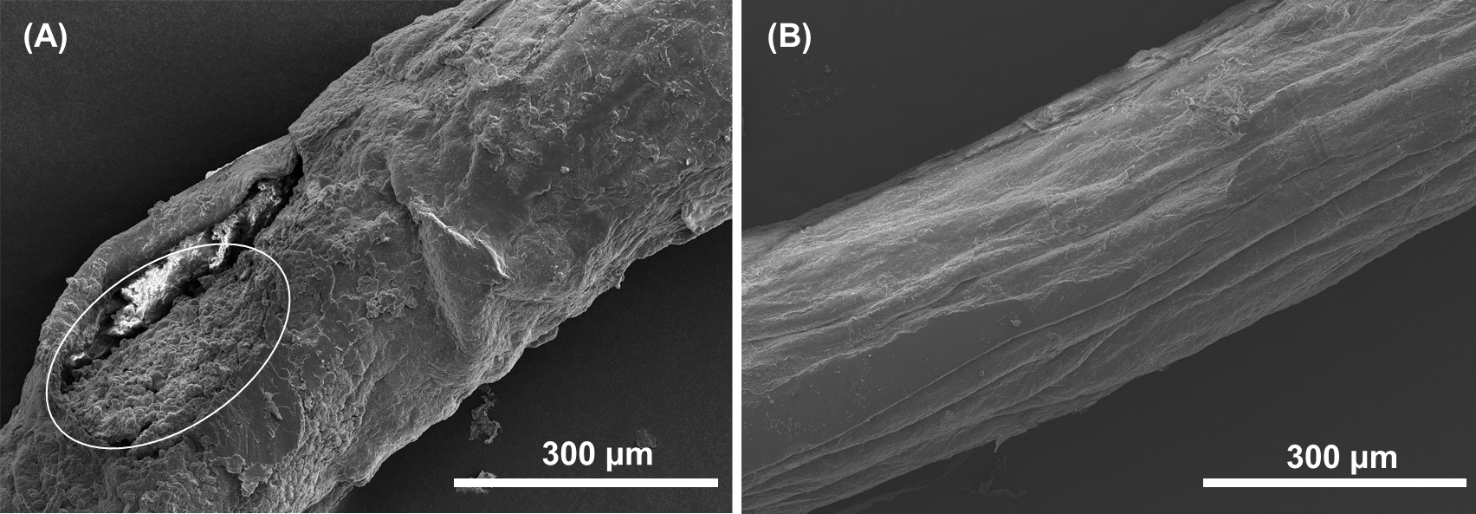
**

**Figure S5.** The GAP-43 expression in optic nerve of RGCs with (A) the control, (B) the ONC, and (C) the PEDOT hydrogel group at Day 21.


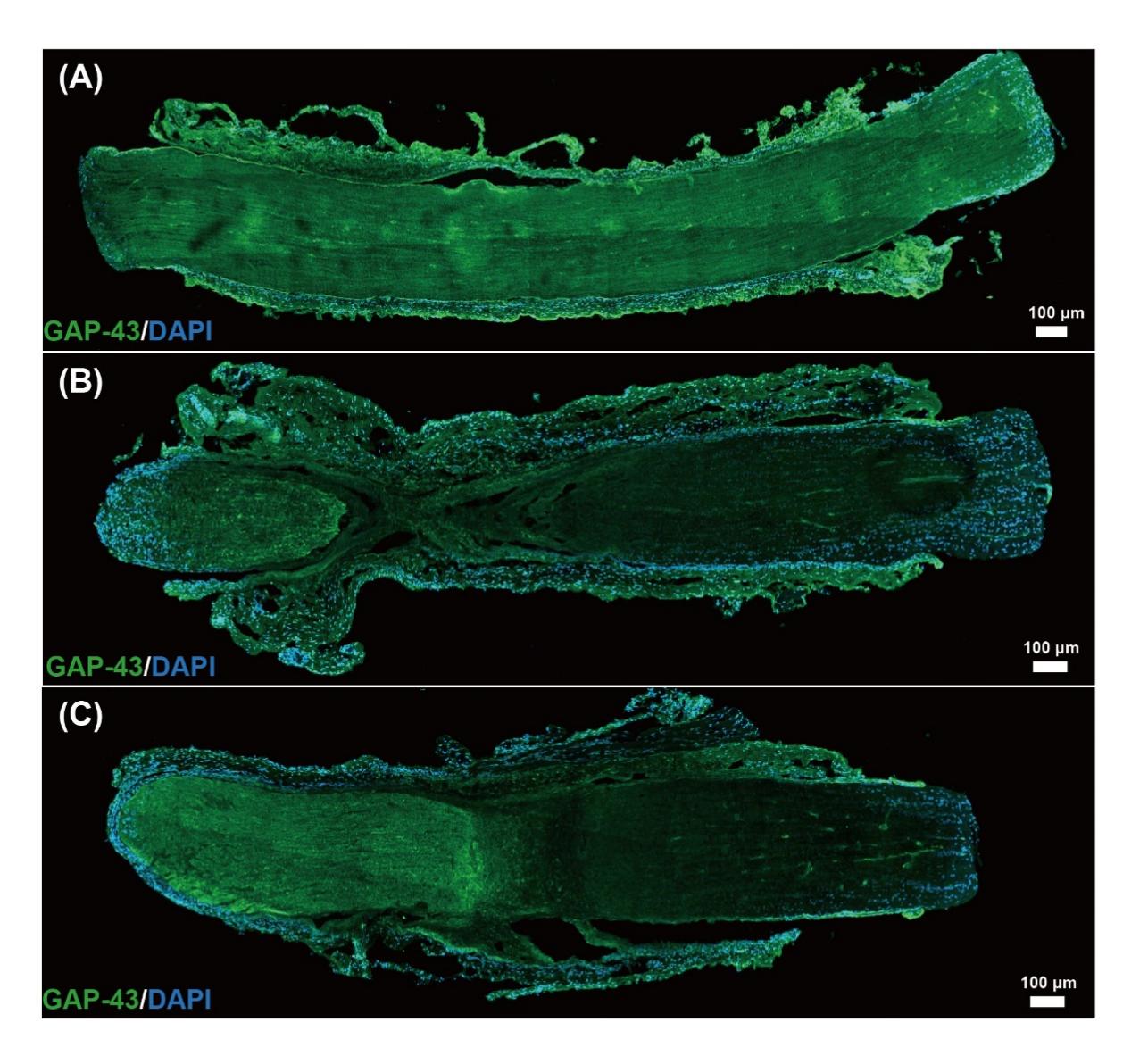


**Figure S6.** The Thy-1 expression in optic nerve of RGCs with (A) the control, (B) the ONC, and (C) the PEDOT hydrogel group at Day 21. The ONC probably triggers the decline in Thy-1 (white frame).


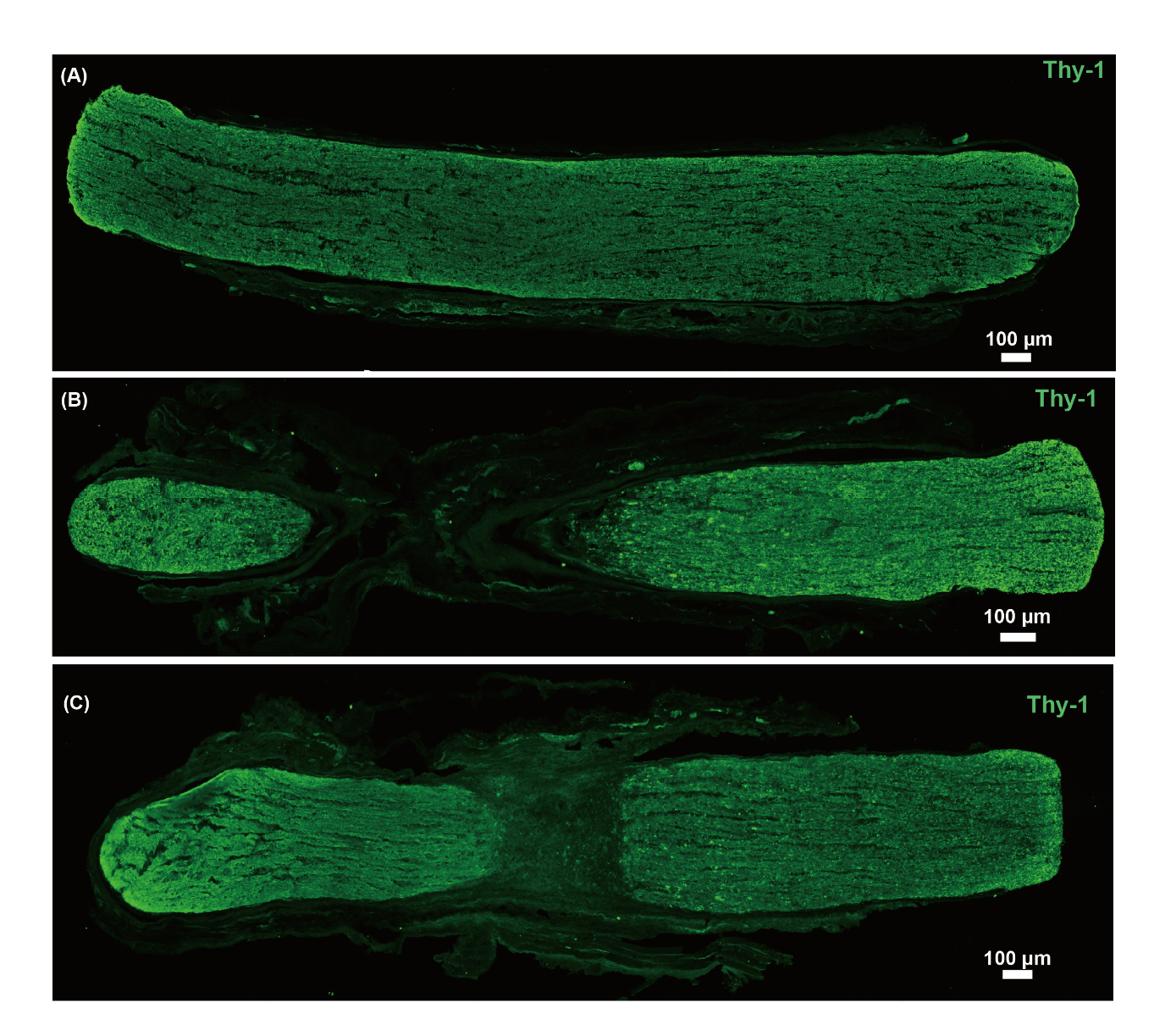


**Figure S7.** Quantitation of RGC survival of the whole RGC population in each group at day 14 (n=4).


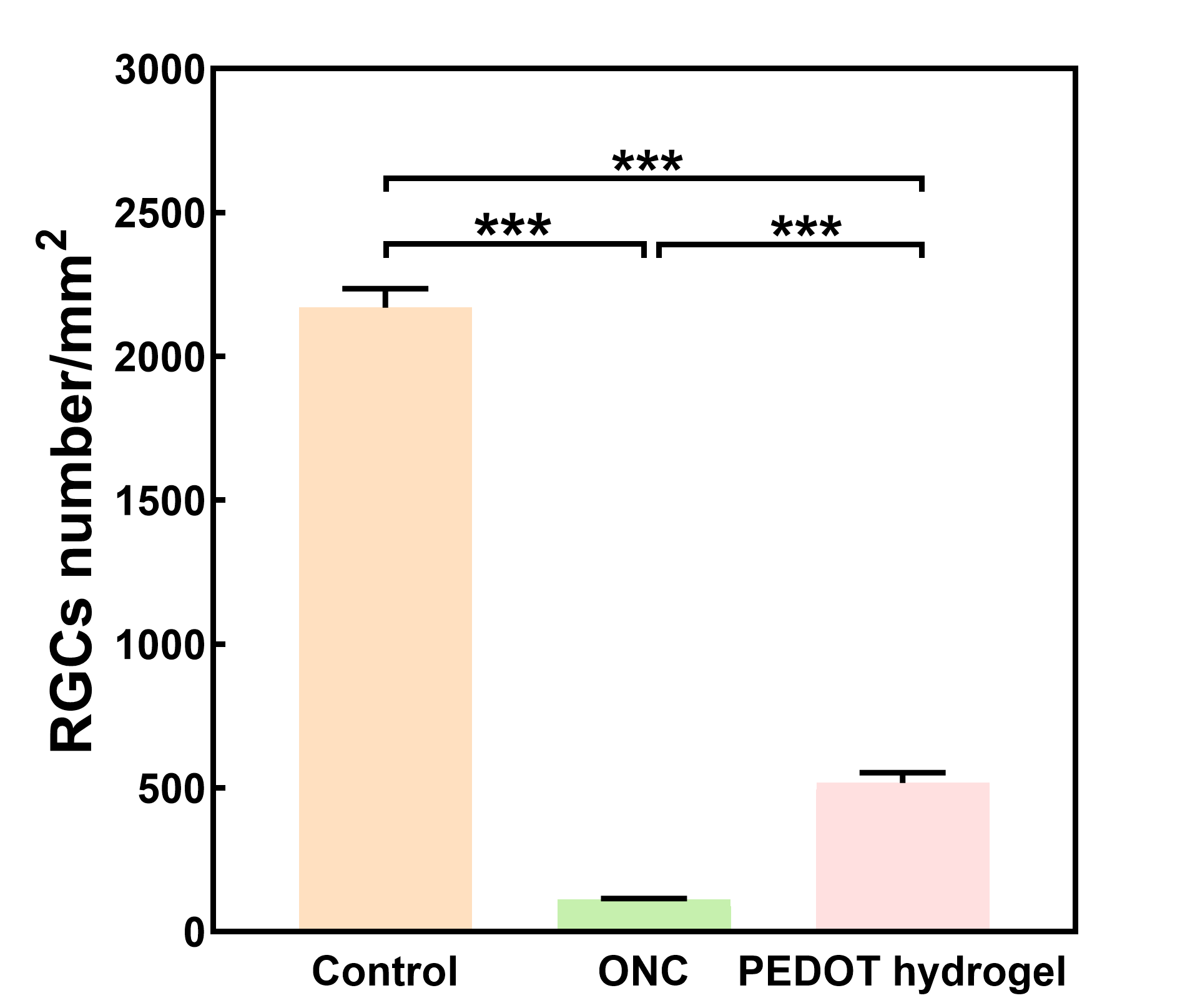


**Figure S8.** Quantitation of RGC survival in the center, paracentral, and peripheral regions in each group at day 14 (n=4).


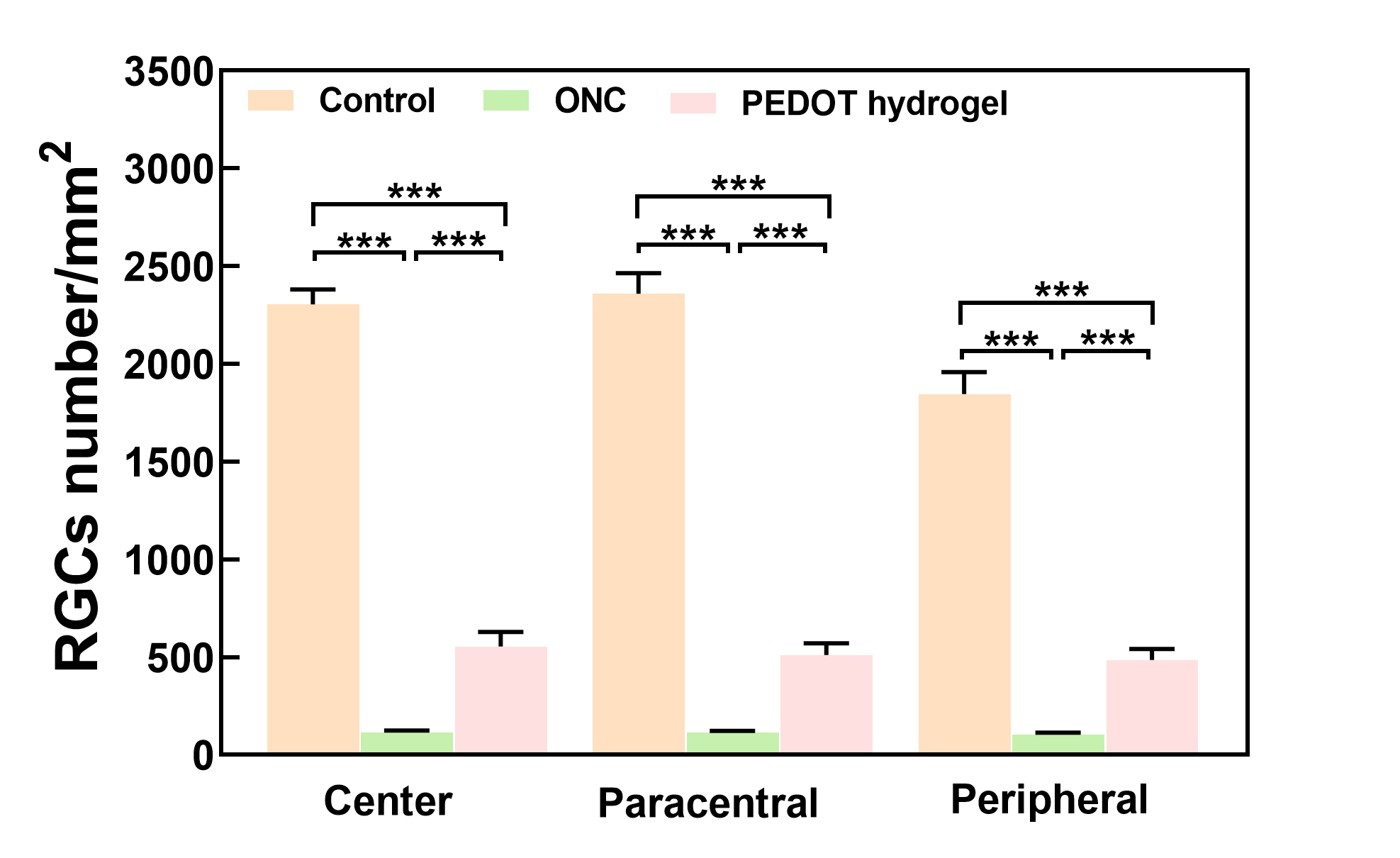


**Figure S9.** (A)Western blot analysis of the expression of GFAP. (B) The picture of the entire PVDF membrane. (C) The original picture of the WB.


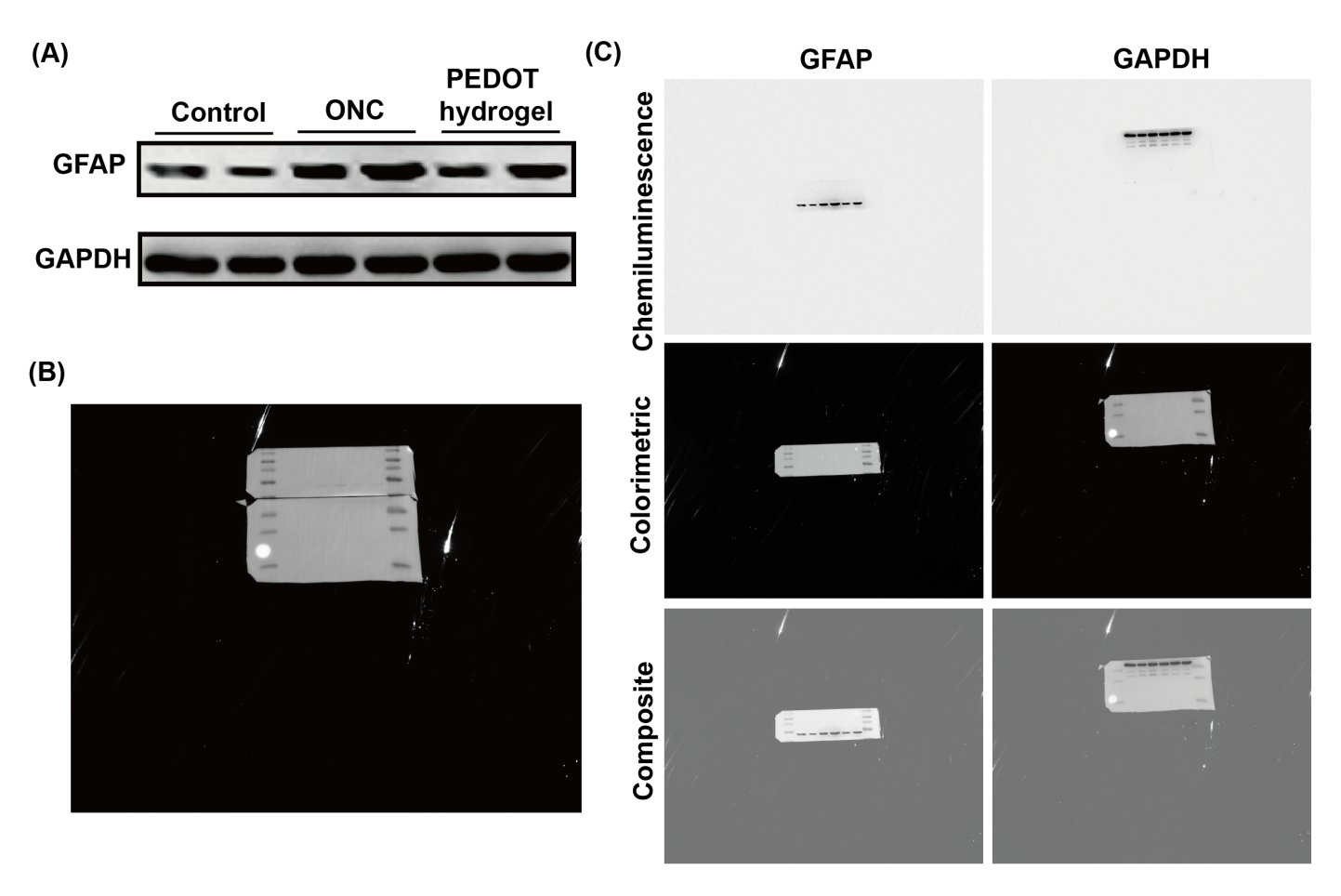


**Figure S10.** The relevant quantitation results obtained from **Figure S9** (n=4).


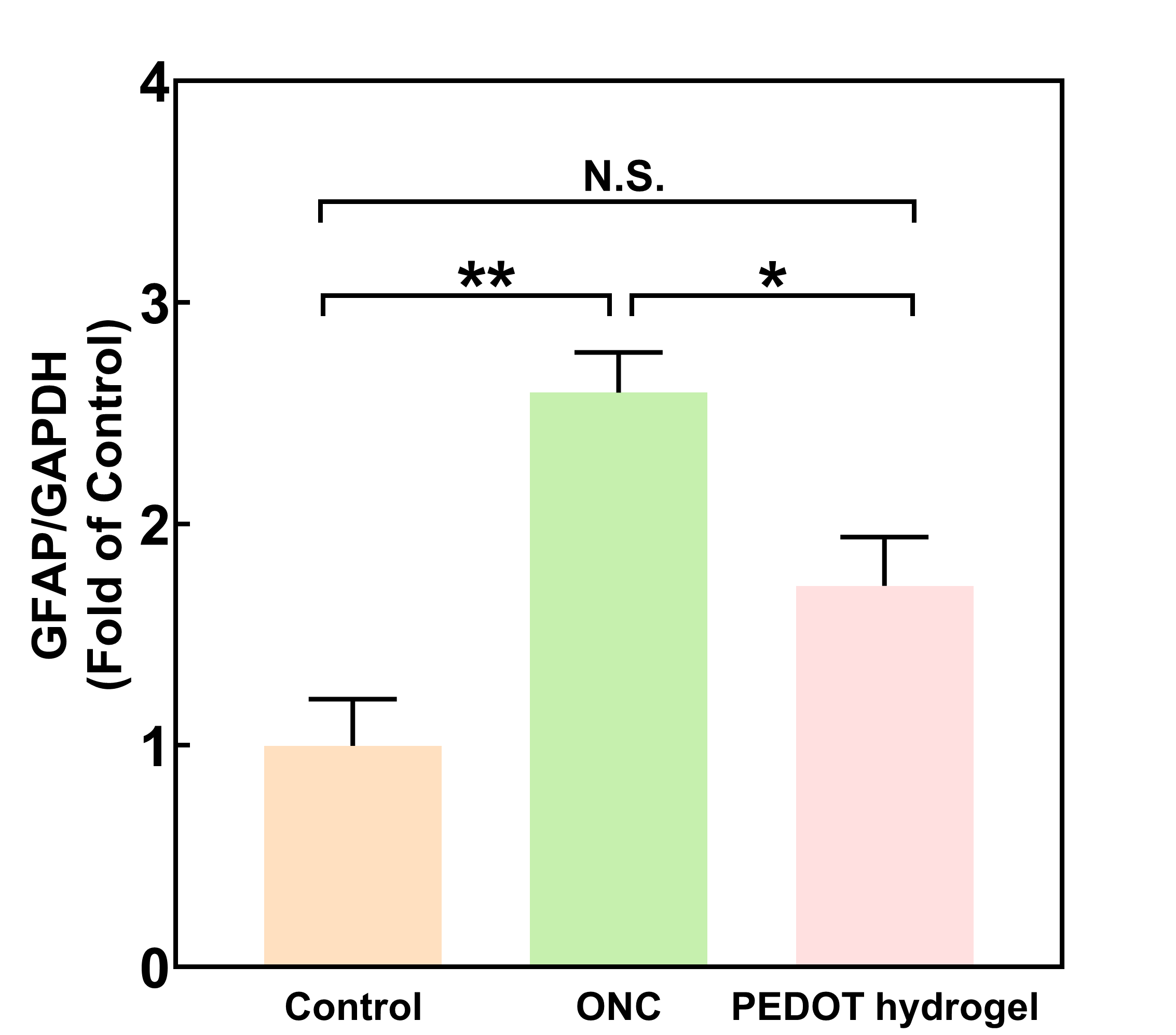


**Figure S11.** Activation of microglia in the outer plexiform layer of retina in different groups at Day 14.


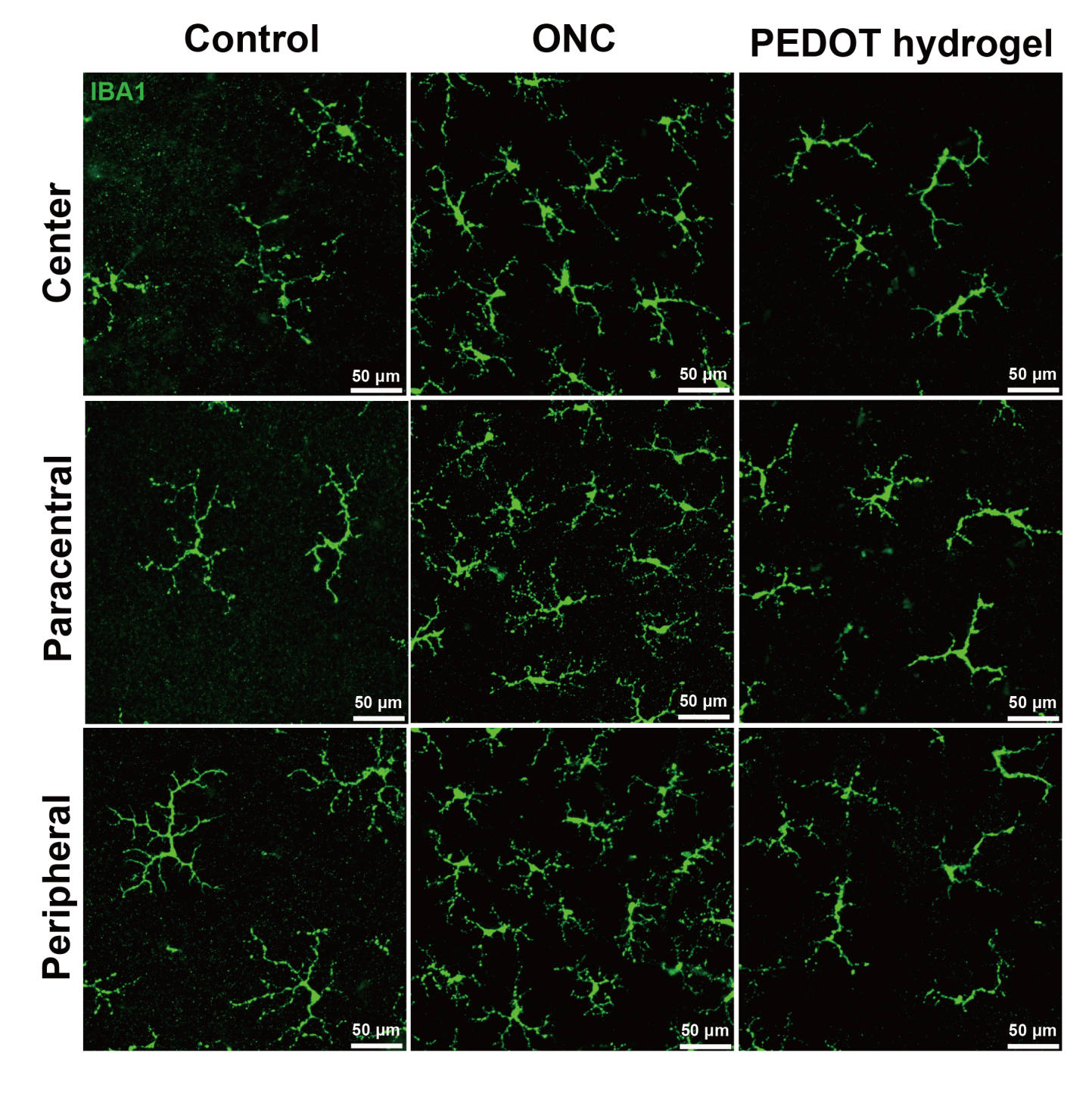


**Figure S12.** Quantitation of microglia number in the outer plexiform layer of retina at the different part at Day 14 (n=4).


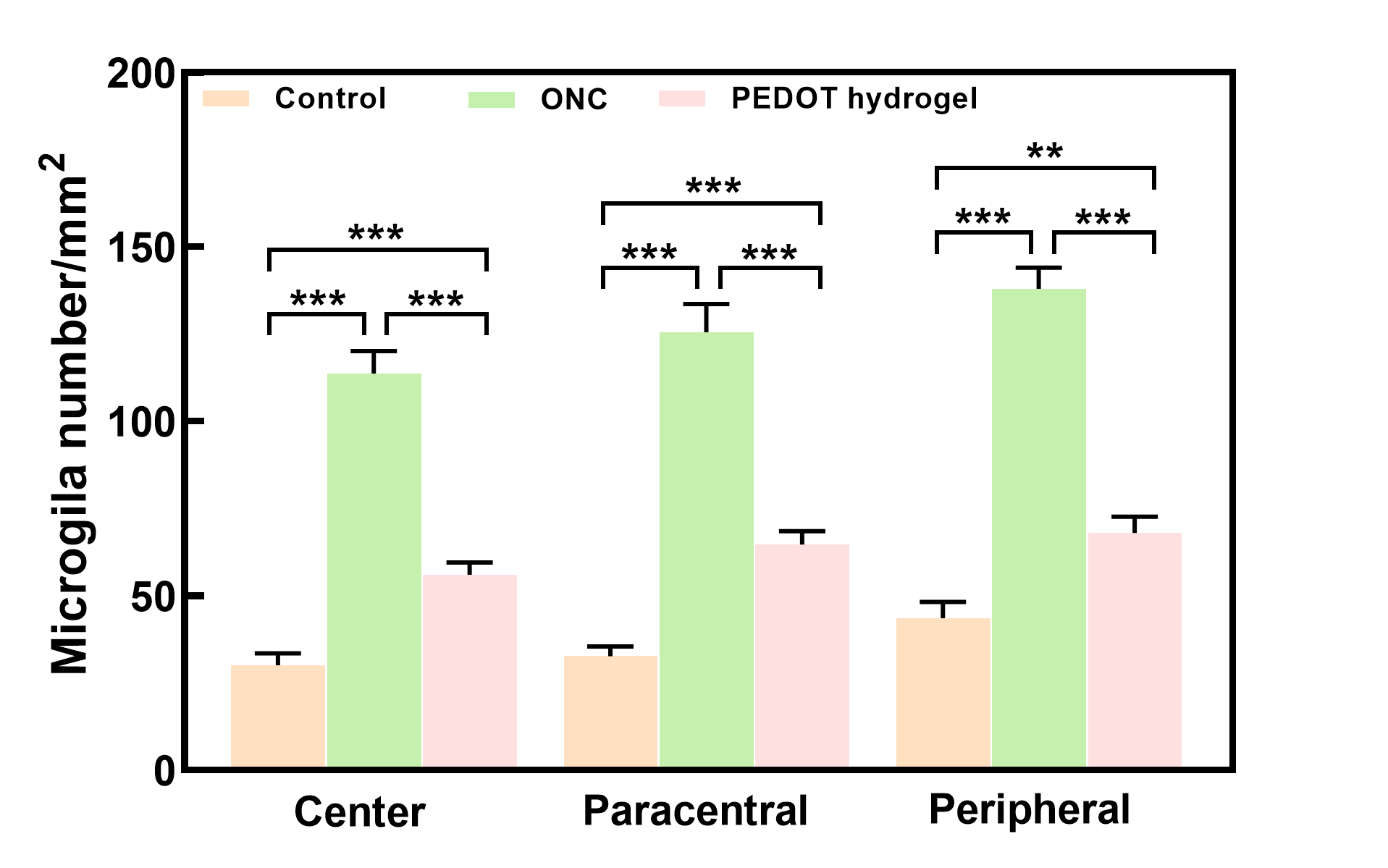


**Figure S13.** Quantitation of microglia number of the whole RGCs in the outer plexiform layer at Day 14 (n=4).


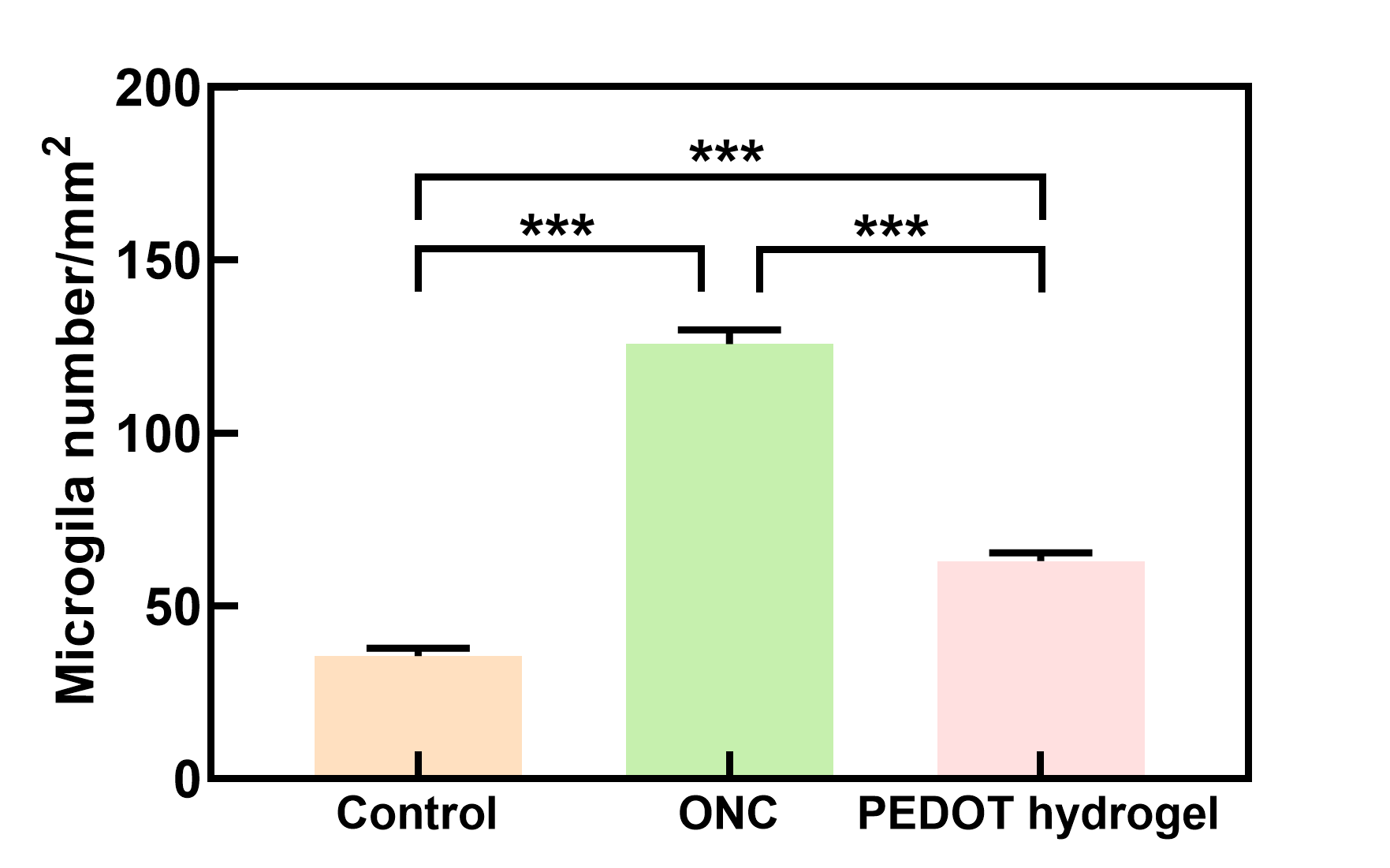


**Figure S14.** Activation of microglia in the ganglion cell layer of retina in different groups at Day 14.


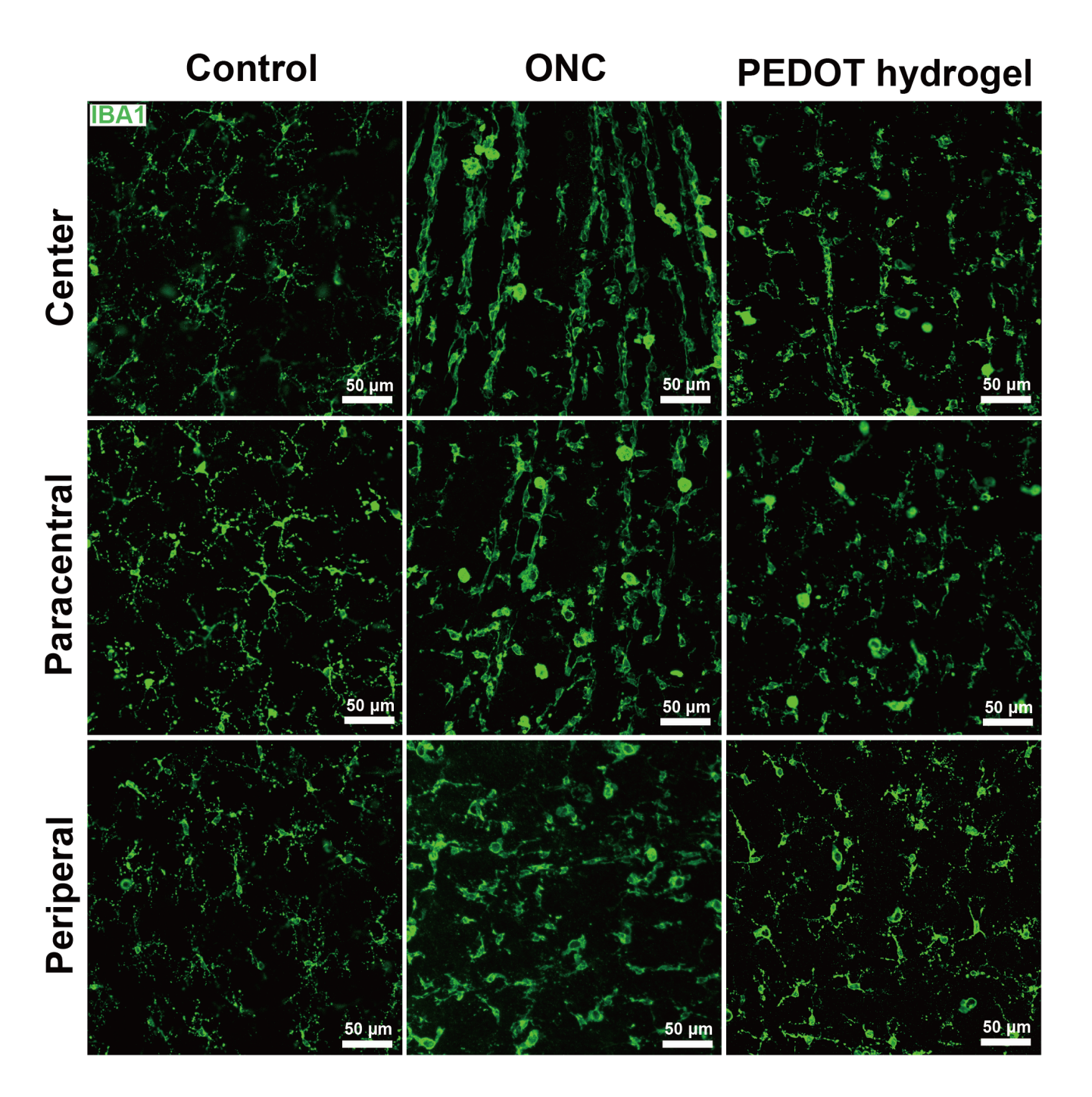


**Figure S15.** Quantitation of microglia number of the center, paracentral, and peripheral part in the ganglion cell layer of the retina at Day 14 (n=4).


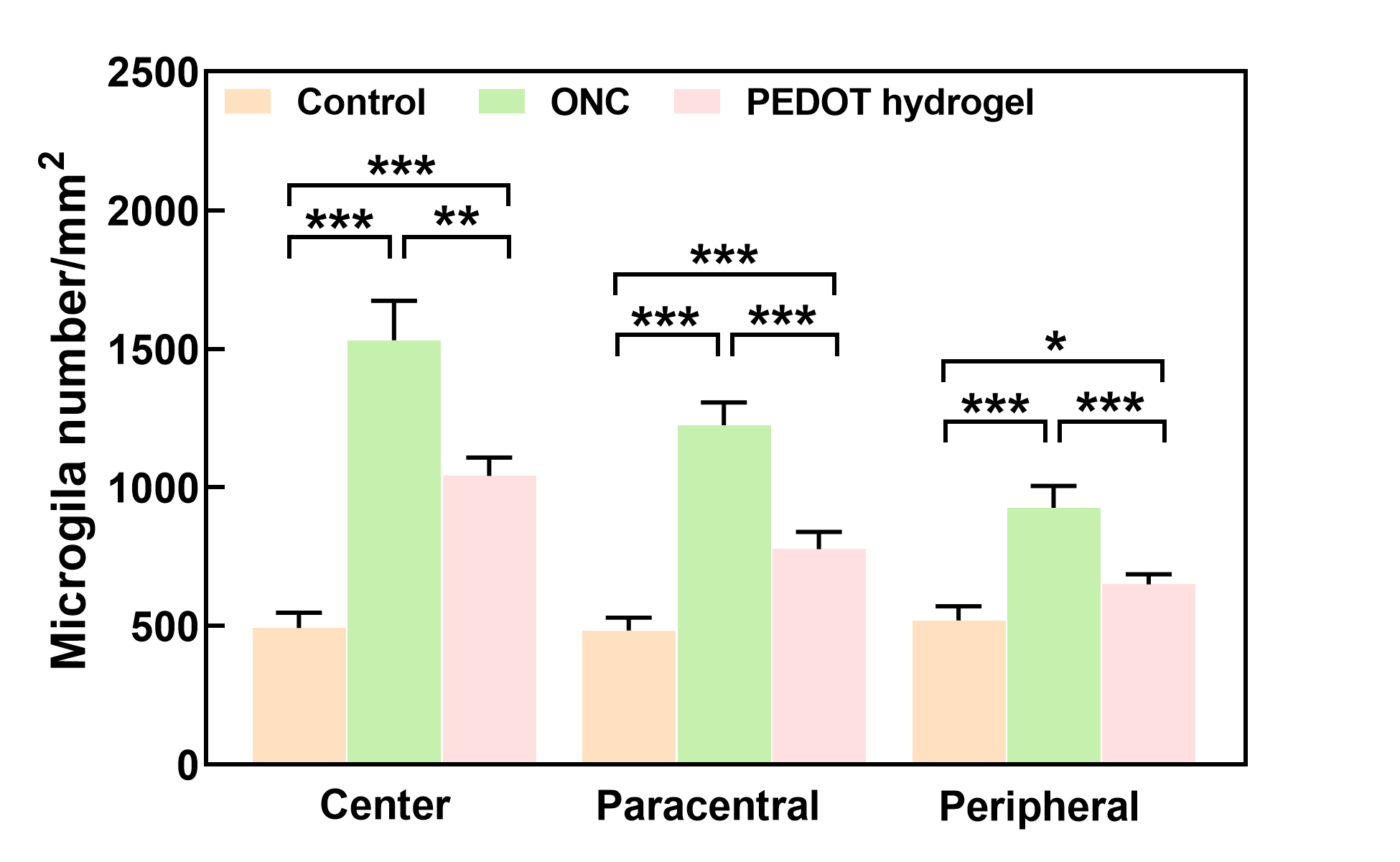


**Figure S16.** Quantitation of microglia number of the whole RGCs in the ganglion cell layer of the retina at Day 14 (n=4).


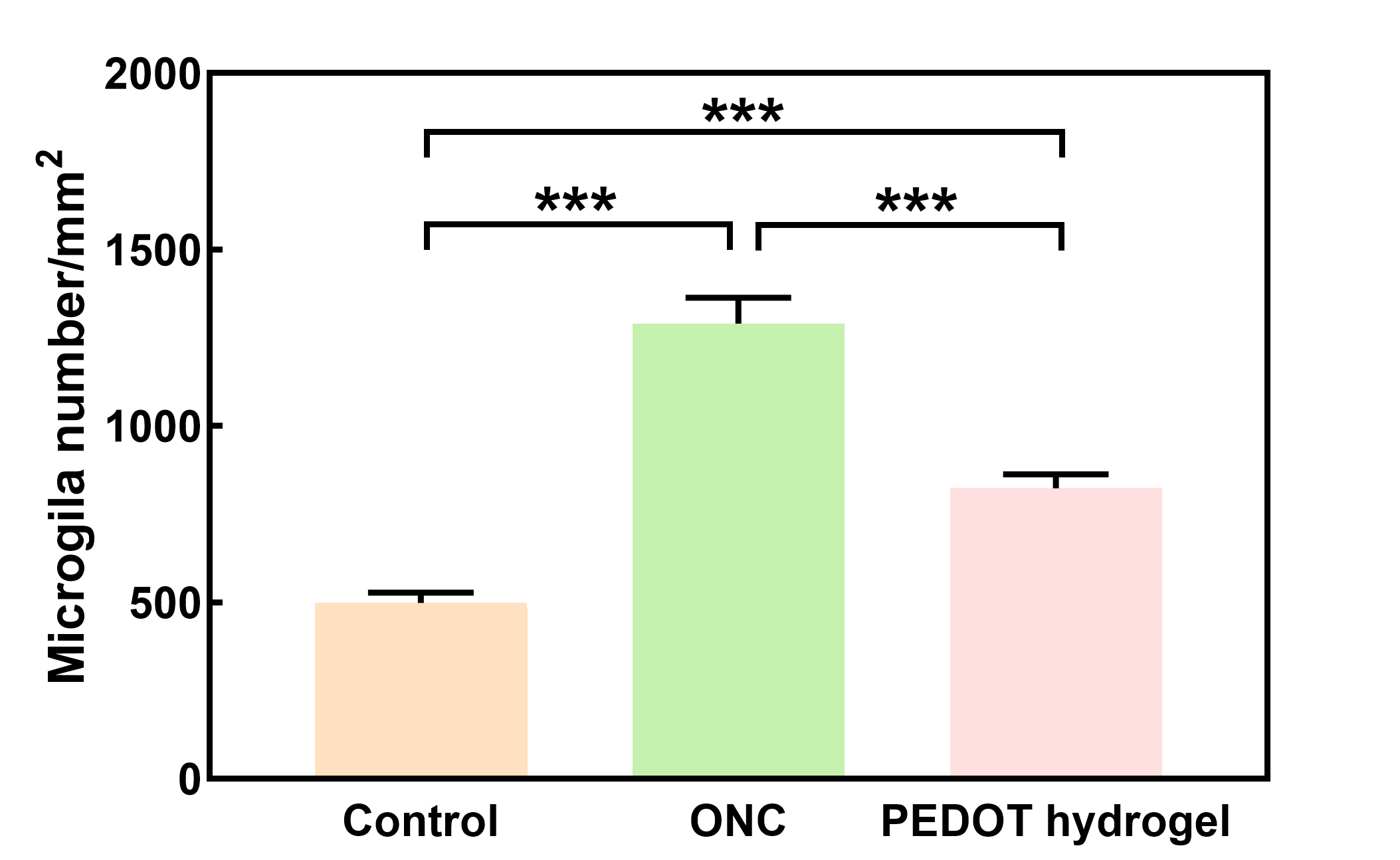


**Figure S17.** The c-fos expression in optic nerve of RGCs with (A) the control, (B) the ONC, and (C) the PEDOT hydrogel group at Day 7.


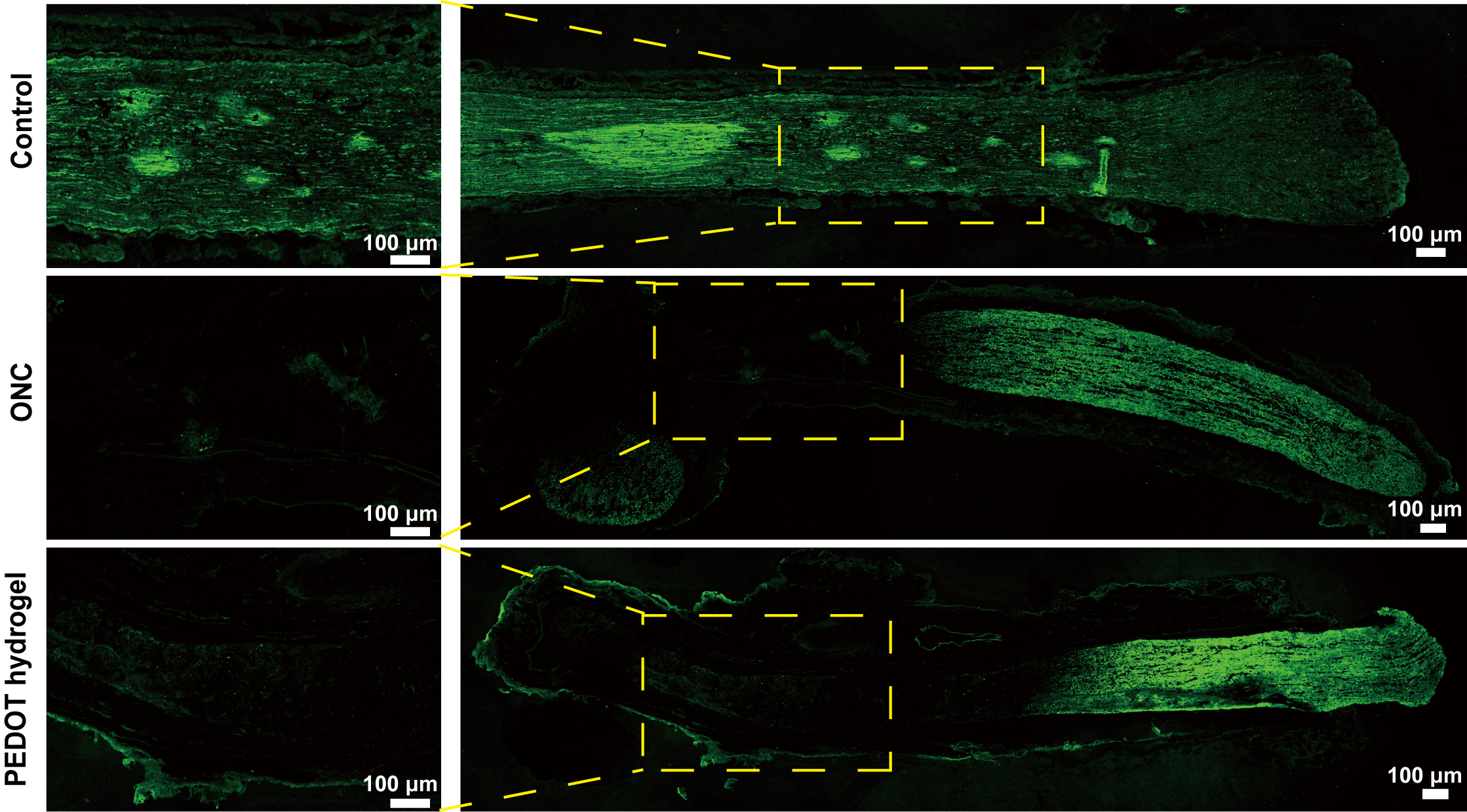

Supplement: Supplementary file 1 — Supporting Information [file ADVS-12-2415601-s001.docx]
